# To move or to evolve: contrasting patterns of intercontinental connectivity and climatic niche evolution in “Terebinthaceae” (Anacardiaceae and Burseraceae)

**DOI:** 10.3389/fgene.2014.00409

**Published:** 2014-11-28

**Authors:** Andrea Weeks, Felipe Zapata, Susan K. Pell, Douglas C. Daly, John D. Mitchell, Paul V. A. Fine

**Affiliations:** ^1^Department of Biology and Ted R. Bradley Herbarium, George Mason UniversityFairfax, VA, USA; ^2^Department of Ecology and Evolutionary Biology, Brown UniversityProvidence, RI, USA; ^3^United States Botanical GardenWashington, DC, USA; ^4^Institute of Systematic Botany, The New York Botanical GardenBronx, NY, USA; ^5^Department of Integrative Biology and Jepson and University Herbaria, University of CaliforniaBerkeley, CA, USA

**Keywords:** biogeography, biome shifts, continental vicariance, diversification, long-distance dispersal, phylogenetic niche conservatism

## Abstract

Many angiosperm families are distributed pantropically, yet for any given continent little is known about which lineages are ancient residents or recent arrivals. Here we use a comprehensive sampling of the pantropical sister pair Anacardiaceae and Burseraceae to assess the relative importance of continental vicariance, long-distance dispersal and niche-conservatism in generating its distinctive pattern of diversity over time. Each family has approximately the same number of species and identical stem age, yet Anacardiaceae display a broader range of fruit morphologies and dispersal strategies and include species that can withstand freezing temperatures, whereas Burseraceae do not. We found that nuclear and chloroplast data yielded a highly supported phylogenetic reconstruction that supports current taxonomic concepts and time-calibrated biogeographic reconstructions that are broadly congruent with the fossil record. We conclude that the most recent common ancestor of these families was widespread and likely distributed in the Northern Hemisphere during the Cretaceous and that vicariance between Eastern and Western Hemispheres coincided with the initial divergence of the families. The tempo of diversification of the families is strikingly different. Anacardiaceae steadily accumulated lineages starting in the Late Cretaceous–Paleocene while the majority of Burseraceae diversification occurred in the Miocene. Multiple dispersal- and vicariance-based intercontinental colonization events are inferred for both families throughout the past 100 million years. However, Anacardiaceae have shifted climatic niches frequently during this time, while Burseraceae have experienced very few shifts between dry and wet climates and only in the tropics. Thus, we conclude that both Anacardiaceae and Burseraceae move easily but that Anacardiaceae have adapted more often, either due to more varied selective pressures or greater intrinsic lability.

## Introduction

A richer understanding of factors underlying the origin of the diverse tropical flora depends on aggregating global biogeographic, taxonomic and phylogenetic information from multiple plant clades. Although much progress has been made reconstructing the tempo and genealogical patterns of angiosperm evolution in the past two decades, most of the major plant lineages lack the comprehensive global sampling and taxonomic studies necessary to address basic questions regarding the timing of radiations, geographic origins, and dispersal histories. Many angiosperm families are common to tropical forests around the world, yet very little is known about which lineages are ancient and have experienced multiple vicariance events, and which lineages have experienced more recent long-distance dispersal. Moreover, the timing and directionality of such dispersal events is often unknown, although recent studies have found that many tropical radiations appear to have coincided with climatic and geographic events, such as Oligocene–Miocene cooling and drying in both the Americas and Africa, the connection of North and South America, and the Andean uplift (Bouchenak-Khelladi et al., 2010; Hoorn et al., 2010; De-Nova et al., 2012; Hughes et al., 2013).

Recent studies have highlighted the importance of phylogenetic niche conservatism in understanding large-scale patterns of plant biogeography and evolution (Wiens and Donoghue, 2004; Donoghue, 2008). The hypothesis is that because adaptation to new climatic zones requires a complex array of morphological and physiological innovations, as long as dispersal is possible, *in situ* adaptation by the resident flora during periods of global cooling or drying may often be less common than immigration of other lineages that are pre-adapted to freezing or dry climates. For example, many Northern American temperate plant lineages have colonized the high-elevation South American Andes since overland connections were established between the two continents (Bell and Donoghue, 2005; Hughes and Eastwood, 2006). However, South American lineages have also colonized these freezing habitats, even from warm tropical lowlands (Donoghue, 2008), demonstrating that adaptation to new biomes, as well as immigration from other areas, can result in diversification within regions (Donoghue, 2008; Donoghue and Edwards, 2014).

**Figure 1:**
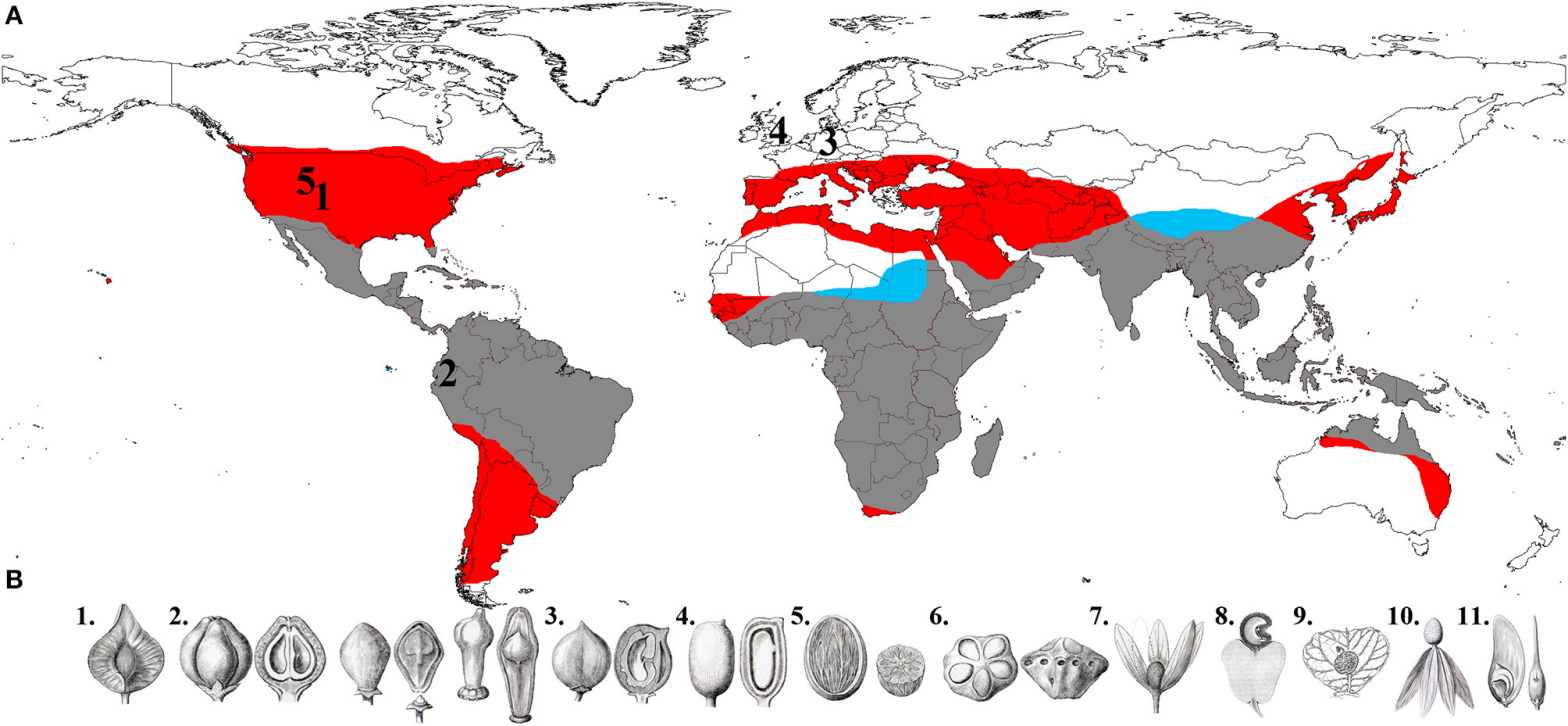
**(A)** Global distribution of Anacardiaceae and Burseraceae (red area is Anacardiaceae only, blue is Burseraceae only, and gray is where the two families' distributions overlap), and fossils used to calibrate the phylogenies in this study (1, Oligocene/Late Eocene *Cotinus* leaf; 2, Middle Miocene *Loxopterygium* fruit; 3, Middle Eocene *Anacardium* fruit; 4, Middle Eocene *Bursericarpum aldwickense* and *Protocommiphora europea* fruits; 5, Middle Eocene *Bursera* subgenus *Elaphrium* leaf). **(B)** Fruit diversity in Anacardiaceae and Burseraceae (1, *Triomma* pseudocapsule*;* 2,* Protium, Bursera* and* Boswellia* nuculaniums; 3, *Garuga* drupe; 4, *Solenocarpus* drupe; 5, *Spondias*, internally operculate drupe; 6, *Dracontomelon*, externally operculate drupe; 7, *Loxostylis*, drupe subtended by wing-like calyx; 8, *Anacardium*, drupe subtended by fleshy hypocarp; 9, *Campylopetalum*, drupe subtended by an accrescent bract; 10, *Swintonia*, drupe subtended by wing-like corolla; 11, *Schinopsis*, samara.) (illustrations 1–7 and 10–11 from Engler, 1883; 8 from Faguet, 1874/1875; 9 copyright Bobbi Angell).

The angiosperm lineages Anacardiaceae and Burseraceae (Sapindales) represent an excellent study system for investigating the biogeographic history of tropical diversification and the relative importance of movement and climatic adaptation in angiosperm evolution. Once recognized as the single taxon “Terebinthaceae” (Marchand, 1869) and subsequently shown to be sister taxa (Fernando et al., 1995; Gadek et al., 1996; Bremer et al., 1999; Savolainen et al., 2000a,b; Pell, 2004), these families collectively comprise ca. 1500 lianas, shrubs and trees distributed on every continent except Antarctica and are major elements of the structure and diversity of temperate, seasonally dry tropical forest and tropical wet forest floras (Gentry, 1988; Pennington et al., 2010) (Figure 1). Anacardiaceae species display a remarkable range of fruit morphologies and seed dispersal syndromes not present in Burseraceae (see Daly et al., 2011; Pell et al., 2011). This disparity is primarily responsible for the recognition of more genera in Anacardiaceae (82) as compared to Burseraceae (19), although each family has approximately the same number of species. However, the geographic range as well as the morphological and ecological diversity of Anacardiaceae considerably eclipses that of Burseraceae, which makes the Terebinthaceae lineage a valuable comparative model system for testing the relative contributions to diversification of climate adaptation and intercontinental movement.

The differences between the families raise the question of how sister lineages having identical ages and nearly equivalent numbers of species could have taken such different evolutionary trajectories whilst becoming so widespread. Anacardiaceae comprise a cosmopolitan group, are found at a greater range of latitudes and elevations, and a broader range of habitats than Burseraceae. All Burseraceae, by contrast, are intolerant of frost and are thus limited to lower elevation zones in the African, American, Asian and Pacific (sub-)tropics.

Testing which events may have been correlated with cladogenesis in Terebinthaceae is contingent on reconstructing a densely sampled, time-calibrated phylogeny for the group. Divergence time estimates are a critical component of testing historical biogeographic hypotheses for Terebinthaceae because routes of overland range expansion available to Anacardiaceae and Burseraceae may have been different as global climate fluctuated over geological time. For example, ancestral lineages of frost tolerant Anacardiaceae may have been able to disperse through regions located above the frost line during the Miocene (e.g., *Rhus*, Yi et al., 2004) whereas the entirely frost-intolerant Burseraceae may have been excluded from them. Historically, angiosperm biogeographers have hypothesized that cosmopolitan and pantropical groups experienced vicariance as a consequence of the break-up of Gondwana (Raven and Axelrod, 1974). Both Anacardiaceae and Burseraceae had been considered Gondwanan families in the past (Raven and Axelrod, 1974; Gentry, 1982). More recently, divergence time estimates for a number of angiosperm lineages having predominantly frost-free distributions, including Burseraceae, have indicated that the northern hemisphere land corridors have had a more influential role in establishing species' ranges than previously hypothesized (Chanderbali et al., 2001; Renner et al., 2001; Davis et al., 2004; Richardson et al., 2004; Weeks et al., 2005; Zerega et al., 2005). In the case of Burseraceae, Weeks et al. (2005) implicated a North American origin of the family followed by Paleocene migration of lineages eastward over the warm North Atlantic land bridge and along the Tethys Seaway to Southeast Asia, as well as early trans-oceanic dispersals to Africa and South America. However, this study did not optimize the ancestral distributions using a geographically diverse group of Anacardiaceae outgroup taxa nor did it incorporate known Anacardiaceae fossils as calibration points.

Contemporary studies have not clarified the relative contribution of vicariance and dispersal in generating the current distribution of both Anacardiaceae and Burseraceae and whether shifts in climatic niches may have promoted their diversification. Here, we address three sets of questions regarding the evolution of Terebinthaceae:

- What is the timing of diversification of Terebinthaceae? Have diversification rates varied through time?
- What is the evolutionary history of geographic range in Terebinthaceae? Have lineages persisted in unique geographic regions or have they dispersed to new geographic regions (i.e., how common is dispersal)?
- What is the evolutionary history of climatic niche (or climatic preference) in Terebinthaceae? Have lineages retained distinct climatic niches or have evolved climatic tolerances (i.e., how common is “niche expansion”)?

## Materials and methods

### Taxon sampling

Ingroup species from Anacardiaceae and Burseraceae were chosen on the basis of assembling the most complete biogeographic coverage possible from all major lineages. Species were selected with reference to preexisting phylogenies (Pell, 2004; Weeks et al., 2005; Fine et al., 2014) as well as to recent taxonomic literature that recognizes the new segregate genus *Poupartiopsis* (Mitchell et al., 2006) and the newly circumscribed genera *Searsia* and *Protorhus* (Moffett, 2007; Pell et al., 2008; Table 1). From Anacardiaceae we obtained 67 of 82 genera (169 species) and sampling was spread across the higher ranks of recently published classifications: (1) the five tribes sensu Mitchell and Mori (1987) as updated by Pell (2004), including Anacardieae (7/8 genera sampled/genera total), Dobineae (2/2), Rhoeae (39/47), Semecarpeae (3/5), Spondiadeae (16/20); and (2) Pell's and Mitchell's (Mitchell et al., 2006) modifications to Takhtajan's (1987) subfamilial system, including Spondioideae (16/20) and Anacardioideae (50/60). From Burseraceae, we obtained 16 of 19 genera (136 spp.) from the five taxonomic alliances sensu Daly et al. (2011): the *Beiselia* alliance (1/1 genus), the *Protium* alliance (3/3 genera), the *Boswellia* alliance (2/2 genera), the *Bursera* alliance (3/3 genera) and the *Canarium* alliance (7/10 genera). Burseraceae includes several disjunct genera (*Canarium*, *Commiphora*, *Dacryodes*, *Protium*) whose species are distributed in African, American, Asian, and Pacific regions, and care was taken to sample representatives from each. Outgroup taxa from Sapindales were chosen with reference to Gadek et al. (1996) and comprise species of Meliaceae (1 sp.; Muellner et al., 2006), Rutaceae (5 spp.; Groppo et al., 2008), and Sapindaceae incl. Aceraceae (15 spp.; Harrington et al., 2005).

**Table 1:** **Accession information for sampled taxa**.

**ANACARDIACEAE**
*Abrahamia littoralis* ined., Pell 609 (NY), Madagascar, AY594403, KP055360, AY594434; *Allospondias lakonensis* (Pierre) Stapf, Pell 1035 (NY), Vietnam, KP055186, KP055361, KP055483; *Amphipterygium adstringens* (Schltdl.) Schiede ex Standl., Pendry 845 (E), Mexico, KP055187, AY594583, AY594496; *Anacardium excelsum* (Bertero and Balb. ex Kunth) Skeels, Daly 13970 (NY), Colombia, KP055188, KP055362, KP055484; *Anacardium occidentale* L., Mori 24142 (NY), French Guiana, KP055189, KP055363, AY594497; *Anacardium parvifolium* Ducke, Reserva Ducke (INPA), Brazil, KP055190, KP055364, KP055485; *Anacardium spruceanum* Benth. ex Engl., Esteril INPA2527 (INPA), Brazil, KP055191, KP055365, KP055486; *Antrocaryon amazonicum* (Ducke) B.L.Burtt and A.W.Hill, Mitchell 663 (NY), Brazil, AY594410, AY594584, AY594441; *Apterokarpos gardneri* (Engl.) C.T.Rizzini, Pirani 2586 (NY), Brazil, KP055192, AY594585, AY594498; *Astronium fraxinifolium* Schott, Pendry 505 (E), Bolivia, KP055193, AY594586, AY594542; *Astronium lecointei* Ducke, Reserva Ducke (INPA), Brazil, KP055194, KP055366, KP055487; *Baronia taratana* Baker, Pell 625 (NY), Madagascar, KP055195, AY594627, AY594568; *Blepharocarya depauperata* Specht, Craven et al. 6762 (MO), Australia, KP055196, KP055367, KP055488; *Blepharocarya involucrigera* F. Muell., R. Jensen 00826 (A), Australia, KP055197, KP055368, KP055489; *Bonetiella anomala* (I. M. Johnst.) Rzed., Johnston Wendt and Chiang 11488 (F), Mexico, KP055198, AY594587, AY594543; *Bouea macrophylla* Griff., Gentry and Frankie 66957 (NY), Peninsular Malaysia, KP055199, AY594589, AY594500; *Bouea oppositifolia* (Roxb.) Meisn., Ambri and Arifin W746 (A), Papua New Guinea, KP055200, –, KP055490; *Buchanania glabra* Wall. ex Engl., Pell 1062 (NY), Vietnam, KP055201, –, KP055491; *Buchanania reticulata* Hance, Pell 1057 (NY), Vietnam, KP055202, KP055369, KP055492; *Buchanania siamensis* Miq., Pell 1054 (NY), Vietnam, KP055203, KP055370, KP055493; *Campnosperma gummiferum* (Benth.) Marchand, Reserva Ducke (INPA), Brazil, KP055204, KP055371, KP055494; *Campnosperma micranteium* Marchand, Randrianaivo 691 (MO), Madagascar, KP055205, KP055372, KP055495; *Campnosperma schatzii* Randrian. and J.S. Mill., Randrianasolo et al. 602 (MO), Madagascar, KP055206, KP055373, –; *Campylopetalum siamense* Forman, Garrett 1398 (NY), Vietnam, KP055207, KP055374, KP055496; *Cardenasiodendron brachypterum* (Loes.) F.A.Barkley, Pendry 691 (E), Bolivia, KP055208, KP055375, AY594503; *Choerospondias axillaris* (Roxb.) B.L. Burtt and A.W. Hill, S. K. Pell 1108 (NY), Vietnam, KP055209, KP055376, KP055497; *Comocladia dodonaea* (L.) Urban, Specht 10 (NY), Puerto Rico, KP055210, AY594592, KP055498; *Comocladia engleriana* Loes., Garcia Castaneda 1472 (LL), Mexico, KP055211, KP055377, AY594506; *Comocladia mayana* Atha J.D. Mitch. and Pell, Atha 5604 (NY), Belize, KP055212, KP055378, KP055499; *Comocladia mollissima* Kunth, Gillis I0317 (TEX), Mexico, KP055213, KP055379, KP055500; *Cotinus coggygria* Scop., Bamps 8753 (LSU), France, KP055214, –, AY594545; *Cotinus obovata* Raf., Reichard 386 (MOR), USA, KP055215, AY594593, AY594546; *Cyrtocarpa edulis* (Brandegee) Standl., Elias 10714 (F), Mexico, KP055216, KP055380, AY594547; *Cyrtocarpa procera* Kunth, Torres 1240 (NY), Mexico, KP055217, AY594596, AY594548; *Dobinea vulgaris* Buch.-Ham., Delendick 76.1570 (NY), Nepal, KP055218, –, AY594512; *Dracontomelon dao* (Blanco) Merr. and Rolfe, Pell 807 (BKL), USA (cultivated in Hawaii), KP055219, KP055381, KP055501; *Dracontomelon duperreanum* Pierre, Pell 1034 (NY), Vietnam, KP055220, KP055382, KP055502; *Dracontomelon vitiense* Engl., Regaldo and Vodonaivalu 905 (F), Fiji, KP055221, KP055383, AY594550; *Drimycarpus racemosus* (Roxb.) Hook. f. 1, Grierson/Long 4261 (A), Bhutan, KP055222, –, KP055503; *Drimycarpus racemosus* (Roxb.) Hook. f. 2, Pell 1118 (NY), Vietnam, KP055223, KP055384, KP055504; *Euroschinus aoupiniensis* Hoff., Pell 1134 (BKL), New Caledonia, KP055224, KP055385, KP055505; *Euroschinus elegans* Engl., J. Munzinger 6642 (BKL), New Caledonia, KP055225, KP055386, KP055506; *Euroschinus falcata* Hook.f., Herscovitch s.n. (NY), Australia, KP055226, KP055387, KP055507; *Euroschinus jaffrei* M. Hoff, McPherson 18174 (MO), New Caledonia, KP055227, KP055388, KP055508; *Euroschinus papuana* Merr. and L.M.Perry, Takeuchi et al. 16409 (A), Papua New Guinea, KP055228, KP055389, KP055509; *Euroschinus verrucosus* Engl., Guillaumin et al. 12227 (NY), New Caledonia, KP055229, KP055390, KP055510; *Euroschinus vieillardii* Engl. var. *glabra*, Pell 1140 (NY), New Caledonia, KP055230, KP055391, KP055511; *Faguetia falcata* Marchand, Pell 600 (NY), Madagascar, KP055231, AY594598, KP055512; *Fegimanra africana* (Oliv.) Pierre, Reitsma and Reitsma 1257 (MO), Gabon, KP055232, AY594599, AY594515; *Fegimanra afzelii* Engl., G. Walters 647 (MO), Gabon, KP055233, KP055392, KP055513; *Gluta renghas* L., Pell 806 (BKL), Malaysia, KP055234, KP055393, KP055514; *Gluta tavoyana* Hook. f., Pell 1075 (NY), Vietnam, KP055235, KP055394, –; *Gluta tourtour* Marchand, Randrianasolo 770 (MO), Madagascar, KP055236, KP055395, KP055515; *Gluta wallichii* (Hook. f.) Ding Hou, Beaman 7065 (NY), Borneo, KP055237, AY594600, AY594516; *Haplorhus peruviana* Engl., O. Zöllner 4030 (L), Chile, KP055238, KP055396, KP055516; *Harpephyllum caffrum* Bernh. ex Krauss, Lau 1588 (NY), USA (cultivated in Hawaii), KP055239, AY594601, AY594518; *Heeria argentea* Meisn., Goldblatt s.n. (MO), South Africa, KP055240, AY594602, KP055517; *Lannea coromandelica* (Houtt.) Merr., Pell 1041 (NY), Vietnam, KP055241, KP055397, KP055518; *Lannea rivae* (Chiov.) Sacleux, Randrianasolo 662 (MO), Tanzania, KP055242, KP055398, AY594520; *Lannea schweinfurthii* Engl., Randrianasolo 661 (MO), Tanzania, KP055243, AY594605, AY594552; *Lannea welwitschii* (Hiern) Engl., Nemba and Thomas 532 (NY), Cameroon, KP055244, KP055399, AY594553; *Laurophyllus capensis* Thunb., Brand 207 (NY), South Africa, KP055245, KP055400, KP055519; *Lithrea molleoides* (Vell.) Engl., Pendry 711 (E), Bolivia, KP055246, KP055401, AY594554; *Loxopterygium grisebachii* Hieron., Pendry 678 (E), Bolivia, KP055247, KP055402, KP055520; *Loxopterygium sagotii* Hook.f., Polak 309 (E), Guyana, KP055248, AY594606, KP055521; *Loxostylis alata* Spreng. ex Rchb., Mitchell 652 (NY), South Africa, KP055249, AY594607, AY594522; *Mangifera foetida* Lour., Pell 1097 (NY), Vietnam, KP055250, KP055403, KP055522; *Mangifera minor* Blume., Pell 982 (NY), Papua New Guinea, KP055251, KP055404, –; *Mauria heterophylla* Kunth, Woytkowski 7788 (G), Peru, KP055252, KP055405, KP055523; *Mauria simplicifolia* Kunth, Leiva et al. 1552 (F), Peru, KP055253, KP055406, AY594556; *Mauria thaumatophylla* Loes., Nee and Wee 53816 (NY), Bolivia, KP055254, KP055407, KP055524; *Melanochyla angustifolia* Hook. f., AC Church 312 (A), Indonesia, KP055255, KP055408, KP055525; *Melanochyla bracteata* King, Niyomdham 1174 (A), Thailand, KP055256, KP055409, KP055526; *Melanochyla castaneifolia* Ding Hou, Ambriansyah and Arifin 903 (L), Indonesia, KP055257, KP055410, KP055527; *Metopium brownei* Urb., Brokaw 295 (NY), Belize, KP055258, AY594609, AY594557; *Metopium toxiferum* (L.) Krug and Urb., P. Fine s.n. (UC), Cuba, –, –, KP055528; *Micronychia bemangidiensis* Randrian. and Lowry, Birkinshaw 1622 (MO), Madagascar, KP055259, KP055411, KP055529; *Micronychia macrophylla* H. Perrier, Pell 643 (NY), Madagascar, AY594414, AY594610, AY594443; *Micronychia tsiramiramy* H. Perrier, Pell 634 (NY), Madagascar, –, AY594611, AY594524; *Myracrodruon balansae* (Engl.) Santin, Schinini 24043 (F), Paraguay, KP055260, KP055412, AY594559; *Myracrodruon urundeuva* Allem., Pendry 724 (E), Bolivia, KP055261, AY594613, AY594560; *Ochoterenaea colombiana* F.A. Barkley, Sánchez 2598 (F), Colombia, KP055262, KP055413, AY594561; *Operculicarya decaryi* H. Perrier, Randrianasolo 627 (MO), Madagascar, KP055263, AY594614, AY594525; *Operculicarya pachypus* Eggli, Pell 664 (NY), Madagascar, KP055264, KP055414, KP055530; *Orthopterygium huaucui* (A. Gray) Hemsl., Smith 5726 (NY), Peru, KP055265, AY594615, AY594526; *Ozoroa dispar* (C. Presl) R. Fern. and A. Fern., R. Brand 33 (NY), South Africa, KP055266, KP055415, KP055531; *Ozoroa insignis* Delile, Randrianasolo 680 (MO), Tanzania, AY594415, KP055416, AY594444; *Ozoroa mucronata* (Bernh.) R. Fern. and A. Fern., R. Brand 1078 (NY), South Africa, KP055267, KP055417, KP055532; *Ozoroa obovata* (Oliv.) R. Fern. and A. Fern., Randrianasolo 707 (MO), Tanzania, AY594416, –, AY594445; *Ozoroa pulcherrima* (Schweinf.) R.Fern. and A. Fern., Luwiika et al. 305 (BRIT), Zambia, KP055268, KP055418, KP055533; *Pegia nitida* Colebr., Zhanhuo 92-254 (MO), China, KP055269, KP055419, AY594563; *Pegia sarmentosa* (Lecomte) Hand.-Mazz., Pell 1096 (NY), Vietnam, KP055270, KP055420, KP055534; *Pentaspadon annamense* (Evrard and Tardieu) P.H. Hô, S. K. Pell 1042 (NY), Vietnam, KP055271, KP055421, KP055535; *Pentaspadon poilanei* (Evrard and Tardieu) P.H. Hô, S. K. Pell 1036 (NY), Vietnam, KP055272, KP055422, KP055536; *Pistacia atlantica* Desf., Frantz s.n. (BRIT), USA (cultivated), KP055273, KP055423, KP055537; *Pistacia chinensis* Bunge, Heng 11622 (CAS), China, KP055274, KP055424, KP055538; *Pistacia mexicana* Kunth, Calzada 20869 (BRIT), Mexico, KP055275, KP055425, KP055539; *Pistacia vera* L., Pell 304 (LSU), USA (cultivated), KP055276, KP055426, KP055540; *Pistacia weinmannifolia* J. Poiss. ex Franch., Pell 1098 (NY), Vietnam, KP055277, KP055427, KP055541; *Pleiogynium hapalum* A. C. Sm., Smith 1940 (G), Fiji, KP055278, KP055428, KP055542; *Pleiogynium timoriense* (A. DC.) Leenh., PIF28193 (A), Queensland, KP055279, KP055429, KP055543; *Poupartia minor* Marchand, Pell 657 (NY), Madagascar, KP055280, KP055430, AY594530; *Poupartiopsis spondiocarpus* Capuron ex J.D. Mitch. and Daly, Randrianasolo 592 (MO), Madagascar, KP055281, KP055431, AY594446; *Protorhus grandidieri* Engl., Randrianasolo 1230 (MO), Madagascar, KP055282, KP055432, KP055544; *Protorhus longifolia* Engl., Brand 322 (NY), South Africa, KP055283, KP055433, KP055545; *Protorhus sericea* Engl., Randrianasolo 783 (MO), Madagascar, AY594406, KP055434, AY594437; *Protorhus viguieri* H. Perrier, Randrianasolo 776 (MO), Madagascar, KP055284, KP055435, AY594440; *Pseudosmodingium andrieuxii* Engl. 2, Tenorio 17041 (F), Mexico, KP055286, –, AY594565; *Pseudosmodingium andrieuxii* Engl. 1, Tenorio 17041 (F), Mexico, KP055285, KP055436, AY594566; *Pseudospondias microcarpa* Engl., Randrianasolo 809 (MO), Gabon, KP055287, KP055437, KP055546; *Rhus aromatica* Aiton, Mayfield 2881 (LSU), USA, AY594418, AY594621, KP055547; *Rhus chinensis* Mill. 1, S. K. Pell 1063 (NY), Vietnam, KP055288, KP055438, KP055548; *Rhus chinensis* Mill. 2, Altvatter and Hammond 7132 V95 (MOR), USA (cultivated from Japan), KP055289, AY594622, KP055549; *Rhus ciliolata* Turcz., G. Hall 0777 (NY), Mexico, KP055290, KP055439, KP055550; *Rhus copallina* L., Mitchell 666 (NY), USA, AY594419, AY594623, KP055551; *Rhus coriaria* L., E. Vitek 2000-301 (W), Portugal (naturalized), KP055291, KP055440, KP055552; *Rhus glabra* L. 'Laciniata', S.K. Pell 750 (BKL), USA (cultivated), KP055292, KP055441, KP055553; *Rhus lanceolata* (A. Gray) Britton, Campbell 39 (NY), USA (cultivated), KP055293, AY594625, AY594449; *Rhus michauxii* Sarg., living collection accession 080590 (ODU), USA, KP055294, KP055442, KP055554; *Rhus ovata* S. Watson, J. D. Mitchell 1503 (NY), USA, KP055295, KP055443, KP055555; *Rhus perrieri* (Courchet) H.Perrier, Randrianasolo 629 (MO), Madagascar, AY594421, AY594626, KP055556; *Rhus sandwichii* A. Gray, Pell 831 (NY), USA (Hawaii), KP055296, KP055444, KP055557; *Rhus thouarsii* (Engl.) H. Perrier, Pell 638 (NY), Madagascar, KP055297, AY594628, AY594452; *Rhus typhina* L., Mitchell 672 (NY), USA, KP055298, AY594629, AY594453; *Rhus virens* Lindh. ex A. Gray, Mitchell 667 (NY), USA, KP055299, AY594631, KP055558; *Schinopsis brasiliensis* Engl., Bridgewater 1012 (E), UK (cultivated), KP055300, AY594632, KP055559; *Schinopsis marginata* Engl., Nee and Wee 53889 (NY), Bolivia, KP055301, KP055445, –; *Schinus areira* L., Pendry 737 (E), Bolivia, KP055302, AY594633, AY594572; *Schinus fasciculata* (Griseb.) I.M. Johnst., Mendoza 2013 (NY), Bolivia, KP055303, KP055446, KP055560; *Schinus gracilipes* I.M. Johnst., Pell 1008 (BKL), USA (cultivated), KP055304, KP055447, KP055561; *Schinus myrtifolia* (Griseb.) Cabrera, Moraes 1809, Bolivia, KP055305, KP055448, KP055562; *Schinus terebinthifolia* Raddi, Prinzie 111 (MO), USA, KP055306, KP055449, KP055563; *Sclerocarya birrea* Hochst. subsp. *caffra* (Sond.) Kokwaro, SKP 695 (NY), USA (cultivated from South Africa), KP055307, AY594634, AY594574; *Searsia erosa* (Thunb.) Moffett, Stevenson 1395170 (NY), South Africa, AY594420, AY594624, AY594448; *Searsia lancea* (L.f.) F.A.Barkley, Pell 693 (BKL), USA (cultivated from South Africa), KP055308, KP055450, KP055564; *Searsia longipes* (Engl.) Moffett, A. Randrianasolo et al. 675 (MO), Tanzania, KP055309, KP055451, KP055565; *Searsia lucida* (L.) F.A.Barkley, Pell 691 (BKL), USA (cultivated from South Africa), KP055310, KP055452, KP055566; *Searsia pendulina* (Jacq.) Moffett, Pell 694 (BKL), USA (cultivated from South Africa), KP055311, KP055453, AY594450; *Searsia undulata* (Jacq.) T.S.Yi A.J.Mill. and J.Wen, Pell 692 (BKL), USA (cultivated from South Africa), AY594423, AY594630, AY594454; *Semecarpus anacardium* L. f., Codon and Codon 13 (NY), Nepal, KP055312, AY594635, AY594575; *Semecarpus forstenii* Blume, Regalado and Sirikolo 812 (F), Solomon Islands, KP055313, KP055454, AY594535; *Semecarpus magnificus* K. Schum., Tree OE4C0215 (MIN), Papua New Guinea, KP055314, KP055455, KP055567; *Semecarpus neocaledonicus* Engl., Pell 1128 (NY), New Caledonia, KP055315, KP055456, KP055568; *Semecarpus obscurus* Thwaites, Motley 2914 (NY), Mauritius, KP055316, KP055457, KP055569; *Semecarpus reticulatus* Lecomte, Pell 1084 (NY), Vietnam, KP055317, KP055458, KP055570; *Semecarpus schlechteri* Lauterb., Tree WP3B0619 (MIN), Papua New Guinea, KP055318, KP055459, KP055571; *Semecarpus tonkinensis* Lecomte, S. K. Pell 1094 (NY), Vietnam, KP055319, KP055460, KP055572; *Smodingium argutum* E. Mey., Winter 88 (MOR), South Africa (cultivated), KP055320, AY594636, AY594576; *Sorindeia juglandifolia* (A. Rich.) Planch. ex Oliv., G. Walters 875 (MO), Gabon, KP055321, KP055461, KP055573; *Spondias malayana* Kosterm., Pell 775 (BKL), USA (cultivated in Hawaii), KP055322, KP055462, KP055574; *Spondias mombin* L., Mitchell s.n. (NY), USA (cultivated), –, –, KP055575; *Spondias pinnata* (Linn. f.) Kurz, Pell 1060 (NY), Vietnam, KP055323, KP055463, KP055576; *Spondias tuberosa* Arruda, W. Thomas s.n. (NY), Brazil, KP055324, KP055464, KP055577; *Swintonia schwenckii* Teijsm. and Binn. ex Hook. f., Herscovitch s.n. (NY), Australia (cultivated), KP055325, KP055465, KP055578; *Tapirira bethanniana* J.D. Mitch., Mori 24337 (NY), French Guiana, KP055326, AY594638, AY594578; *Tapirira guianensis* Aubl. 2, Cornejo and Canga 8194 (NY), Ecuador, KP055328, KP055467, KP055580; *Tapirira guianensis* Aubl. 1, Daly 13984 (NY), Colombia, KP055327, KP055466, KP055579; *Tapirira obtusa* (Benth.) J.D. Mitch., Mori 24744 (NY), French Guiana, KP055329, AY594639, AY594579; *Thyrsodium spruceanum* Benth., Mori 24215 (NY), French Guiana, KP055330, AY594641, –; *Toxicodendron borneense* (Stapf) Gillis, Sidiyasa and Arifin 1481 (L), Indonesia, KP055331, –, –; *Toxicodendron griffithii* (Hook. f.) Kuntze, Koelz 30428 (L), India, KP055332, –, –; *Toxicodendron pubescens* Mill., Mitchell 1501 (NY), USA, KP055333, KP055468, KP055582; *Toxicodendron radicans* (L.) Kuntze, Pell 545 (LSU), USA, KP055334, AY594642, AY594540; *Toxicodendron rhetsoides* (Craib) Tardieu, Maxwell 90-101 (L), Thailand, KP055335, –, –; *Toxicodendron succedaneum* (L.) Kuntze, Pell 1092 (NY), Vietnam, KP055336, KP055469, KP055583; *Toxicodendron vernicifluum* (Stokes) F.A. Barkley, Mitchell 660 (NY), USA (cultivated from South Korea), KP055337, AY594643, AY594580; *Toxicodendron vernix* (L.) Kuntze, Mitchell 673 (NY), USA, KP055338, KP055470, AY594581; *Trichoscypha acuminata* Engl., Walters et al. 539 (MO), Gabon, AY594425, KP055471, AY594456; *Trichoscypha ulugurensis* Mildbr., Randrianasolo 726 (MO), Tanzania, AY594426, –, AY594457.
**BURSERACEAE**
*Ambilobea madagascariensis* (Capuron) Thulin, Beier and Razafim., Nusbaumer LN905 (MO), Madagascar, KF034990, KM516857, –; *Aucoumea klaineana* Pierre, Walters et al. 466 (MO), Gabon, FJ233911, KM516858, GU246086; *Beiselia mexicana* Forman, Pell s.n. (NY), Mexico, AY315111-2, AY314997, GU246085; *Boswellia frereana* Birdw., Thulin and Warfa 5599 (UPS), Somalia, AY315084-6, AY314998, KM516800; *Boswellia neglecta* S. Moore, Weeks 00-VIII-29-1 (TEX), Ethiopia, AY315087-9, AY314999, GU246087; *Boswellia sacra* Birdw. (syn = *B*. *carteri*), Weeks 01-X-08-3 (TEX), North East Africa, AY315090-2, AY315000, GU246088; *Bursera biflora* Standl., Weeks 99-VII-17-7 (TEX), Mexico, AY315039-41, AY315001, GU246089; *Bursera copallifera* (Sessé and Moc. ex DC.) Engl., Weeks 00-X-24-1 (TEX), Mexico, AY315042-4, AY315002, KM516801; *Bursera coyucensis* Bullock, Weeks 98-VII-15-3 (TEX), Mexico, KM516830, KM516859, –; *Bursera cuneata* (Schltdl.) Engl., Weeks 99-VII-17-1 (TEX), Mexico, AY315045-7, AY315003, GU246090; *Bursera discolor* Rzed., Weeks 98-VII-15-1 (TEX), Mexico, AY309305-7, AY309282, KM516802; *Bursera fagaroides* (H.B.K.) Engl., Weeks 01-X-08-1 (TEX), Mexico, AY309308-10, AY309283, KM516803; *Bursera hindsiana* Engl., Weeks 00-VI-14-1 (TEX), Mexico, AY315048-50, AY315004, GU246091; *Bursera infernidialis* F.Guevara-Fefer and Rzed., Weeks 99-X-11-1 (TEX), Mexico, KM516831, KM516860, KM516804; *Bursera lancifolia* (Schltdl.) Engl., Weeks 98-VII-14-5 (TEX), Mexico, AY309317-20, AY309286, GU246092; *Bursera longipes* Standl., Weeks 98-VII-14-6 (TEX), Mexico, AY309320-2, AY309287, –; *Bursera microphylla* A. Gray, Weeks 01-X-08-2 (TEX), USA, AY309326-8, AY309289, GU246093; *Bursera penicillata* Engl., Weeks 99-X-13-2 (TEX), Mexico, KM516832, KM516861, KM516805; *Bursera sarukhanii* Guevara and Rzed., Weeks 00-VIII-18-6 (TEX), Mexico, AY315051-3, AY315005, KM516806; *Bursera simaruba* (L.) Sarg., Goldman s.n. (BH), USA, AY309341-3, AY309293, GU246094; *Bursera spinescens* Urb. and Ekman, Weeks 01-VIII-23-1 (TEX), Dominican Republic, AY309356-8, AY309294, KM516807; *Bursera steyermarkii* Standl., Weeks 99-VI-13-5 (TEX), Guatemala, KM516833, KM516862, –; *Bursera tecomaca* (DC.) Standl., Weeks 02-IV-23-1 (TEX), Mexico, AY309359-61, AY309280, FJ466463; *Canarium album* (Lour.) Raeusch., HCAN 24/N98-18 at NGR, China, AY635362, AY635355, FJ466464; *Canarium balansae* Engl., Munzinger 2965 (NOU), New Caledonia, FJ466459, FJ466493, FJ466465; *Canarium bengalense* Roxb., HCAN 25/N98-19 at NGR, China, AY635363, AY635356, FJ466466; *Canarium decumanum* Gaertn., HCAN 6/N90-155 at NGR, Malaysia, AY635364, AY635357, FJ466467; *Canarium harveyi* Seem., HCAN 16/N92-30 at NGR, unknown, AY635365, AY635358, FJ466468; *Canarium indicum* L., Lai s.n. (BH), Malaysia, AY315113-5, AY315006, FJ466469; *Canarium madagascariense* Engl., Randrianaivo et al. 746 (MO), Madagascar, FJ466462, FJ466496, FJ466471; *Canarium madagascariense* subsp. *bullatum* Leenh., Daly 12952 (NY), Madagascar, KM516834, KM516863, KM516808; *Canarium muelleri* F.M.Bailey, Fine 1400 (NY), Australia, KM516836, KM516865, GU246095; *Canarium obtusifolium* Scott-Elliot, Daly 12953 (NY), Madagascar, KM516837, KM516866, KM516810; *Canarium oleiferum* Baill., Munzinger GD 1373 (NOU), New Caledonia, FJ466460, FJ466494, FJ466472; *Canarium ovatum* Engl., HCAN 7/N91-26 at NGR, Philippines, AY635366-8, AY635359, FJ466473; *Canarium pilosum* A.W. Benn., Bogler s.n. (TEX), Malaysia, AY315119-20, AY315008, FJ466474; *Canarium* sp. nov. 1, Daly 12967 (NY), Madagascar, KM516835, KM516864, KM516809; *Canarium* sp. nov. 2, Daly 12963 (NY), Madagascar, KM516838, KM516867, KM516811; *Canarium strictum* Roxb., HCAN 22/97-02 at NGR, China, AY635369, AY635360, FJ466475; *Canarium tramdenum* C.D. Dai and Yakolvlev, HCAN 23/N97-04 AT NGR, China, AY635370, AY635361, FJ466476; *Canarium vulgare* Leenh., Lai s.n. (BH), Malaysia, AY315121-3, AY315009, FJ466477; *Canarium whitei* Guillaumin, Munzinger LB600 (NOU), New Caledonia, FJ466461, FJ466495, FJ466478; *Canarium zeylanicum* Blume, Lai s.n. (BH), Malaysia, AY315124-6, AY315010, FJ466479; *Commiphora angolensis* Engl., Raal and Raal 801 (TEX), South Africa, AY315054-6, AY315011, KM516812; *Commiphora aprevalii* Guillaumin, Phillipson 2563 (MO), Madagascar, AY831870, AY831942, –; *Commiphora capensis* Engl., Weeks 06-XII-23-1 (GMUF), Namibia, KM516839, KM516868, KM516813; *Commiphora edulis* (Klotzsch) Engl., Weeks 00-VI-14-3 (TEX), Zimbabwe, AY315057-9, AY315012, FJ466480; *Commiphora eminii* subsp. *zimmermannii* (Engl.) J.B. Gillett, Mwandoka and Shangai 595 (MO), Tanzania, AY315060-2, AY315013, KM516814; *Commiphora falcata* Capuron, Phillipson et al. 3744 (MO), Madagascar, AY831875, AY831947, GU246097; *Commiphora franciscana* Capuron, Labat 2082 (MO), Madagascar, AY315063-5, AY315014, KM516815; *Commiphora kua* (R.Br. ex Royle) K. Vollesen, Gilbert et al. 7629 (MO), Ethiopia, AY315066-8, AY315015, –; *Commiphora leptophloeos* (Mart.) J.B. Gillett, Abbott 16295 (TEX), Bolivia, AY315069-71, AY315016, KM516816; *Commiphora monstrosa* (H. Perrier) Capuron, Phillipson 2354 (MO), Madagascar, AY831884, AY831956, –; *Commiphora rostrata* Engl., Gilbert et al. 7472 (MO), Ethiopia, AY315072-4, AY315017, KM516817; *Commiphora saxicola* Engl., Weeks 06-XII-30-1 (GMUF), Namibia, KM516840, KM516869, KM516818; *Commiphora schimperi* Engl., Weeks 00-VIII-18-8 (TEX), South Africa, AY315075-7, AY315018, GU246098; *Commiphora ugogensis* Engl., Lovett 1626 (MO), Tanzania, AY315078-80, AY315019, KM516819; *Commiphora wightii* (Arn.) Bhandari, Weeks 00-VIII-18-3 (TEX), India, AY315081-3, AY315020, KM516820; *Commiphora wildii* Merxm., Weeks 06-XII-30-5 (GMUF), Namibia, KM516841, KM516870, KM516821; *Crepidospermum atlanticum* Daly, Stefano 204 (NY), Brazil, KJ503399, KJ503682, KJ503776; *Crepidospermum rhoifolium* (Benth.) Engl., Daly et al. 13817 (NY), Colombia, KJ503429, KJ503707, KJ503804; *Dacryodes buettneri* H. J. Lam, Carvalho 5748 (TEX), Equatorial Guinea, AY315139-40, AY315024, GU246100; *Dacryodes* cf. *peruviana* (Loes.) H.J.Lam, GV984 (NY), Ecuador, KM516848, KM516871, KM516822; *Dacryodes chimantensis* Steyerm. and Maguire, Fine s.n. (NY), Peru, KM516842, KM516872, KM516823; *Dacryodes cuspidata* (Cuatrec.) Daly, Fine 259 (NY), Peru, KM516843, KM516873, GU246101; *Dacryodes edulis* (G. Don) H.J. Lam, Wilks 2552 (NY), Gabon, KM516844, AY315025, GU246102; *Dacryodes excelsa* Vahl, Struwe and Specht 1085 (NY), Puerto Rico, KM516845, KM516874, AY594509; *Dacryodes hopkinsii* Daly, Fine 137 (NY), Peru, KM516846, KM516875, KM516824; *Dacryodes klaineana* (Pierre) H.J. Lam, Merello et al. 1615 (MO), Equatorial Guinea, AY315141-3, AY315026, KM516825; *Dacryodes nitens* Cuatrec., Fine 1376 (NY), French Guiana, KM516847, KM516876, KM516826; Garuga floribunda Decne., McPherson 19447 (NOU), Malaysia, KM516849, KM516877, GU246105; *Garuga pinnata* Roxb., Maxwell 89-515 (MO), Thailand, KM516850, KM516878, KM516827; *Protium aidanianum* Daly, GV 53 (QCNE), Ecuador, KJ503367, KJ503654, KJ503744; *Protium altsonii* Sandwith, Fine 1298 (UC), Guyana, KJ503346, KJ503635, KJ503720; *Protium amazonicum* (Cuatrec.) Daly, Sara Smith s.n. (UC), Peru, KJ503422, KJ503700, KJ503799; *Protium aracouchini* (Aubl.) Marchand, Fine 1385 (UC), French Guiana, KJ503359, KJ503647, KJ503736; *Protium attenuatum* Urb., Howard 1983 (NY), St. Lucia (Lesser Antilles), KJ503381, KJ503667, KJ503758; *Protium brasiliense* (Spreng.) Engl., MS 227 (NY), Brazil, KJ503404, KJ503687, KJ503781; *Protium calanense* Cuatrec., ND864 (UC), Peru, KJ503379, KJ503665, KJ503756; *Protium calendulinum* Daly, AmaLin tree 19- 111-5 (UC), Peru, KJ503435, KJ503712, KJ503810; *Protium colombianum* Cuatrec., Daly et al. 13819 (NY), Colombia, KJ503430, KJ503708, KJ503805; *Protium confusum* Pittier, Perez 2126 (SCZ), Panama, KJ503342, KJ503632, KJ503716; *Protium copal* (Schltdl. and Cham.) Engl., Daly s.n., Belize, KJ503368, KJ503655, KJ503745; *Protium costaricense* (Rose) Engl., Perez 1984 (SCZ), Costa Rica, KJ503341, KJ503631, KJ503715; *Protium cranipyrenum* Cuatrec., Daly et al. 13831 (NY), Colombia, KJ503433, KJ503711, KJ503808; *Protium crassipetalum* Cuatrec., Fine 1304 (UC), Peru, KJ503356, KJ503643, KJ503730; *Protium cubense* Urb., Fine 2016 (UC), Cuba, KJ503420, KJ503699, KJ503797; *Protium decandrum* (Aubl.) Marchand, Fine 1371 (UC), French Guiana, KJ503376, KJ503663, KJ503753; *Protium demerarense* Swart, Fine 1426 (UC), French Guiana, KJ503353, KJ503641, KJ503727; *Protium divaricatum* Engl. var. *divaricatum*, Fine 292 (UC), Peru, KJ503362, KJ503649, KJ503739; *Protium fragrans* Urb., Fine 2013 (NY), Cuba, KJ503418, KJ503697, KJ503795; *Protium gallosum* Daly, Fine 297 (UC), Peru, KJ503360, KJ503648, KJ503737; *Protium giganteum* Engl., Fine 1372 (UC), French Guiana, KJ503377, KJ503664, KJ503754; *Protium glabrescens* Swart, Fine 215 (UC), Peru, KJ503348, KJ503637, KJ503722; *Protium guianense* (Aubl.) Marchand, Fine 1369 (UC), French Guiana, KJ503374, KJ503661, KJ503751; *Protium heptaphyllum* (Aubl.) Marchand subsp. *heptaphyllum*, Stefano 223 (NY), Brazil, KJ503402, KJ503685, KJ503779; *Protium icicariba* (DC.) Marchand, Stefano 222 (NY), Brazil, KJ503401, KJ503684, KJ503778; *Protium javanicum* Burm.f., Chase 2089 (K), Indonesia, KJ503339, KJ503629, KJ503713; *Protium klugii* J.F.Macbr., Fine 955 (UC), Peru, KJ503366, KJ503653, KJ503743; *Protium laxiflorum* Engl., Fine 311 (UC), Peru, KJ503361, –, KJ503738; *Protium madagascariense* Engl., Daly et al. 13092 (NY), Madagascar, KJ503382, KJ503668, KJ503759; *Protium nervosum* Cuatrec., Daly et al. 13815 (NY), Colombia, KJ503428, KJ503706, –; *Protium nodulosum* Swart, Fine 956 (UC), Peru, KJ503363, KJ503650, KJ503740; *Protium opacum* Swart subsp. *opacum*, Fine 957 (UC), Peru, KJ503365, KJ503652, KJ503742; *Protium ovatum* Engl., Fonseca 169 (NY), Brazil, KJ503408, KJ503689, KJ503785; *Protium pallidum* Cuatrec., Fine 958 (UC), French Guiana, KJ503357, KJ503645, KJ503732; *Protium panamense* I.M.Johnst., Perez 1838 (SCZ), Panama, KJ503344, –, KJ503718; *Protium paniculatum* Engl. var. *paniculatum*, Fine 153 (UC), Peru, KJ503364, KJ503651, KJ503741; *Protium pecuniosum* Daly, Aguila 12937 (NY), Costa Rica, KJ503340, KJ503630, KJ503714; *Protium pilosum* (Cuatrec.) Daly, Fine 1452 (UC), French Guiana, KJ503434, –, KJ503809; *Protium pittieri* (Rose) Engl., Garcia 47 (LSCR), Costa Rica, KJ503398, KJ503681, KJ503775; *Protium plagiocarpium* Benoist, Fine 1363 (UC), French Guiana, KJ503370, KJ503657, KJ503747; *Protium polybotryum* (Turcz.) Engl., PACL Assunção 803 (INPA), Brazil, KJ503396, KJ503679, KJ503773; *Protium puncticulatum* J.F.Macbr., Daly et al. 13776 (NY), Brazil, KJ503388, –, KJ503765; *Protium rhyncophyllum* (Rusby) ined., Daly 12163 (NY), Brazil, KJ503383, KJ503669, KJ503760; *Protium sagotianum* Marchand, Fine 1451 (UC), French Guiana, KJ503351, KJ503639, KJ503725; *Protium serratum* (Wall. ex Colebr.) Engl., Daly et al. 13880 (NY), Vietnam, KJ503410, KJ503691, KJ503787; *Protium sessiliflorum* (Rose) Standl., Perez 1910 (SCZ), Panama, KJ503343, KJ503633, KJ503717; *Protium spruceanum* (Benth.) Engl., ND 1181 (UC), Peru, KJ503355, –, KJ503729; *Protium subacuminatum* Swart, Fine 2001 (UC), Cuba, KJ503416, KJ503695, KJ503793; *Protium unifoliolatum* Engl., ND 1202 (NY), Cuba, KJ503378, –, KJ503755; *Protium warmingianum* Marchand, Stefano 232 (NY), Brazil, KJ503405, –, KJ503782; *Santiria apiculata* A.W. Benn., Lai s.n. (BH), Malaysia, AY315127-9, AY315030, FJ466482; *Santiria griffithii* Engl., Lai s.n. (BH), Malaysia, AY315130-2, AY315031, FJ466483; *Santiria trimera* (Oliver) Aubrév., Bradley et al. 1026 (MO), Gabon, KM516851, KM516879, GU246109; *Scutinanthe brunea* Thw., Mohtar 53964 (MO), Sarawak, KM516852, KM516880, –; *Tetragastris balsamifera* (Sw.) Kuntze, Torrens s.n. (UC), Dominican Republic, KJ503409, KJ503690, KJ503786; *Tetragastris catuaba* Soares da Cunha, Piotto 3850 (NY), Brazil, KJ503413, KJ503694, KJ503790; *Tetragastris hostmannii* (Engl.) Kuntze, Cabral 63 (NY), Brazil, KJ503412, KJ503693, KJ503789; *Tetragastris varians* Little, Daly et al. 13822 (NY), Colombia, KJ503432, KJ503710, KJ503807; *Trattinnickia burserifolia* Mart., Daly et al. 9061 (NY), Brazil, KM516853, KM516881, KM516828; *Trattinnickia* cf. *lancifolia* (Cuatrec.) Daly, GV11958 (NY), Ecuador, KM516855, KM516882, KM516829; *Trattinnickia demerarae* Swart, SM 25262 (NY), French Guiana, KM516854, KM516883, GU246111; *Trattinnickia glaziovii* Swart, Gentry and Revilla 69141 (MO), Brazil, AY315136-8, FJ466498, FJ466485; *Triomma malaccensis* Hook.f., Gentry and Tagi 34056 (MO), Malaysia, KM516856, KM516884, GU246112.
**MELIACEAE**
*Trichilia elegans* A. Juss., Nee and Wee 53785 (NY), Bolivia, KP055339, KP055472, KP055584.
**RUTACEAE**
*Boronia denticulata* Sm., 8116105 (BKL), USA (cultivated in NY), KP055340, –, –; *Dictyoloma peruviana* Planch., L. Valenzuela et al. 3260 (BRIT), Peru, KP055341, KP055473, KP055585; *Poncirus trifoliata* (L.) Raf., SKP 697 (BKL), USA (cultivated in NY), KP055342, KP055474, KP055586; *Spathelia bahamensis* Vict., D. S. Correll 46048 (BRIT), Bahama, KP055343, KP055475, KP055587; *Zanthoxylum* sp., Acevedo 11126 (US), French Guiana, KP055344, –, AY594541.
**SAPINDACEAE**
*Acer cissifolium* K. Koch, SKP 698 (BKL), USA (cultivated in NY), KP055345, KP055476, KP055588; *Acer griseum* (Franch.) Pax, SKP 700 (BKL), USA (cultivated in NY), KP055346, KP055477, KP055589; *Acer mandshuricum* Maxim., SKP 699 (BKL), USA (cultivated in NY), KP055347, –, –; *Acer pensylvanicum* L., SKP 696 (BKL), USA (cultivated in NY), KP055348, –, KP055590; *Cupania scrobiculata* Rich., Acevedo 11119 (US), French Guiana, KP055349, AY594595, AY594508; *Dilodendron bipinnatum* Radlk., Acevedo 11129 (US), Bolivia, KP055350, KP055478, AY594510; *Diplokeleba floribunda* N.E. Br., Acevedo 11130 (US), Bolivia, KP055351, –, AY594511; *Dodonaea viscosa* Jacq., Acevedo 11144 (US), Bolivia, KP055352, AY594597, AY594513; *Guioa koelreuteria* (Blanco) Merr., Takeuchi 7123 (NY), Papua New Guinea, KP055353, KP055479, AY594517; *Hypelate trifoliata* Sw., Acevedo 11425 (US), Puerto Rico, KP055354, AY594604, AY594519; *Placioscyphus* sp., Pell 602 (NY), Madagascar, KP055355, –, AY594528; *Sapindus saponaria* L., Zanoni 15476 (NY), Dominican Republi, KP055356, KP055480, AY594534; *Serjania glabrata* Kunth, Acevedo 6553 (US), Bolivia, KP055357, KP055481, AY594536; *Serjania polyphylla* (L.) Radlk., Acevedo s.n. (US), Puerto Rico (cultivated), KP055358, KP055482, AY594537; *Thouinia portoricensis* Radlk., Acevedo 11435 (US), Puerto Rico, KP055359, –, AY594539.

*Each species name is followed by its herbarium voucher, country of origin and GenBank accession numbers for nrDNA ETS, cpDNA rps16 intron, and trnL-F intron-spacer region. –, DNA sequence missing*.

### Marker selection and sequence alignment

Sequence data for assessing the individual phylogenies of Anacardiaceae and Burseraceae have been generated by current authors using multiple phylogenetic markers (Weeks, 2003; Pell, 2004; Fine et al., 2005, 2014; Weeks et al., 2005; Pell et al., 2008). The published datasets overlapped for three DNA sequence regions: the nuclear ribosomal external transcribed spacer (*ETS*), the chloroplast *trnL* intron and *trnL-F* intergenic spacer (*trnL-F* region), and the chloroplast *rps16* intron. All of these regions have proven alignable across the targeted taxa and useful for investigating phylogeny at the familial and generic levels. These three datasets were expanded with additional taxa for the current study using amplification and sequencing protocols as outlined in publications referenced above. Multiple sequence alignment for each locus was carried out in MAFFT v7.0 (Katoh and Standley, 2013) with the E-INS-i algorithm. To improve alignment quality, we ran GBlocks V0.91b (Castresana, 2000) with parameters −b3 = 4, −b4 = 10, −b5 = h to clean the alignments as this has been shown to improve subsequent phylogenetic analyses (Talavera and Castresana, 2007). Before phylogenetic inference, we evaluated whether the final concatenated matrix should be partitioned by marker or by any combination of markers, and which nucleotide substitution model should be employed for the final partition scheme. For this analysis, we used the Bayesian Information Criterion as implemented in PartitionFinder (Lanfear et al., 2012) using the greedy algorithm, and we unlinked branch length estimates for each of the substitution models in each partition. Results of this analysis showed that the matrix should be treated as a single partition evolving under the GTR+I+Gamma model of nucleotide substitution.

### Sources of fossil calibration points

**Figure 2:**
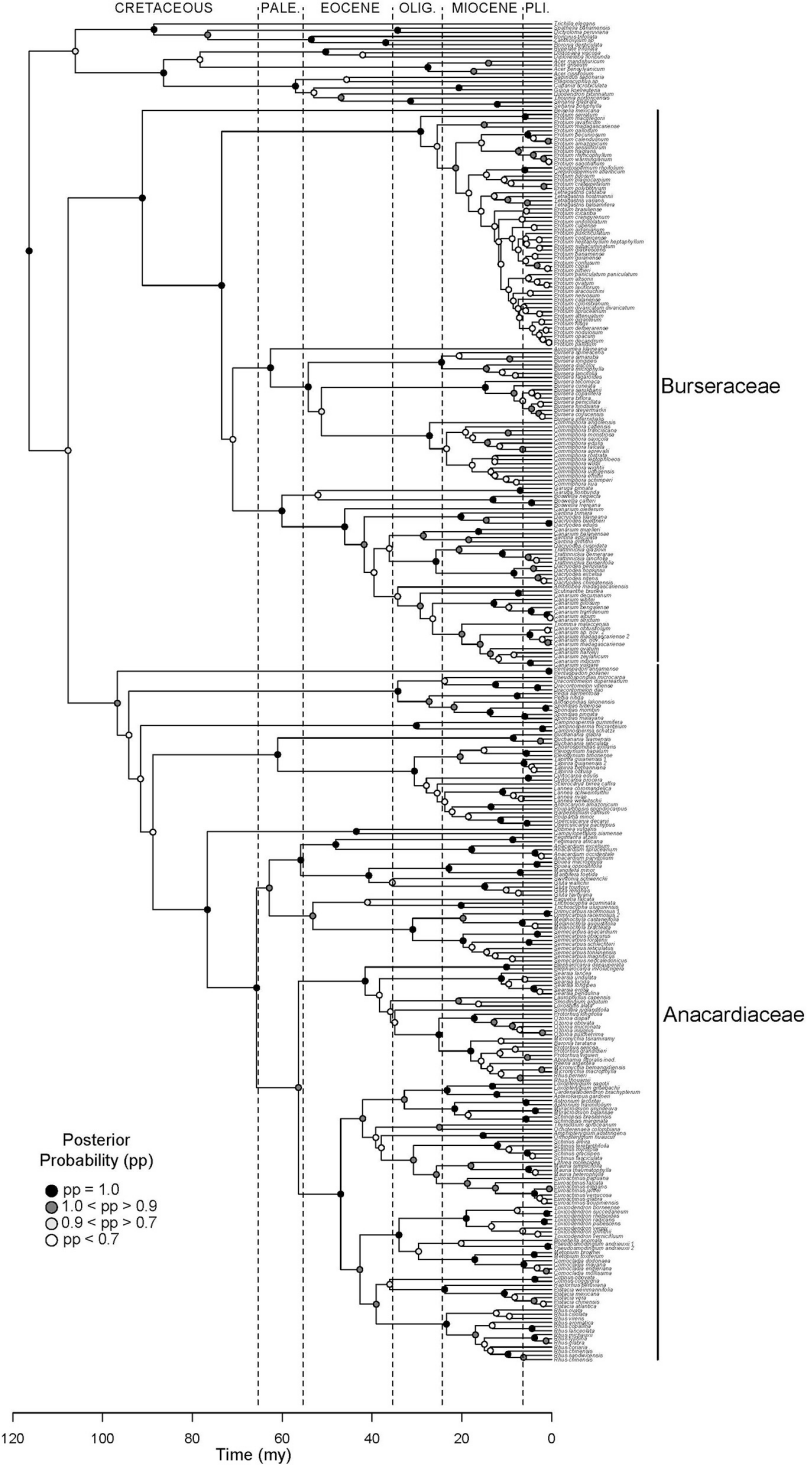
**Molecular phylogeny of Terebinthaceae**. Maximum clade credibility tree (MCCT) summarizing results of Bayesian dating analysis. Families Anacardiaceae and Burseraceae indicated to the right of the clades. Posterior probabilities for branches are shown on descendant nodes.

Both families have rich micro- and macro-fossil records with which to calibrate their phylogeny and test hypotheses of historical biogeographical evolution in Terebinthaceae. The three Anacardiaceae fossils chosen for calibration are classified within extant lineages: (1) an early Oligocene/Late Eocene *Cotinus* leaf fossil from the Florissant flora, Colorado, United States (34 Ma; MacGinitie, 1953); (2) a Middle Miocene *Loxopterygium* fruit fossil from the Ecuadorian Andes (10 Ma; Burnham and Carranco, 2004); and (3) a Middle Eocene *Anacardium* fruit fossil from Germany (47 Ma; Manchester et al., 2007). Ages of all Anacardiaceae fossils included herein are associated with sediments that have been dated radiometrically. Three Burseraceae fossils were selected. Two fossils are from the London Clay and are Early Eocene fruit casts of *Bursericarpum aldwickense* Chandler, which is assignable to extant Protieae on the basis of the number of pyrenes per fruit (Chandler, 1961; Harley and Daly, 1995), and *Protocommiphora europea* Reid and Chandler, which is similar to extant *Commiphora* (Reid and Chandler, 1933; Collinson, 1983). The age of these fossils is estimated as 48.6 Ma, the age of the lowest stratum of the Middle Eocene (Lutetian) and the upper-most bound of the Early Eocene (Ypresian). The remaining Burseraceae fossil is a leaf impression ascribed to *Bursera* subgenus *Elaphrium* from the Green River Flora of Colorado and Utah (MacGinitie, 1969; Plate 30, Figure 2), whose base has a radiometrically-determined age of 49.7–50.7 Ma (Clyde et al., 1995). Dates for all geological periods and epochs follow those of the International Commission on Stratigraphy.

### Phylogenetic dating and diversification analyses

The chronogram and divergence times were co-estimated using Markov Chain Monte Carlo (MC2) sampling in BEAST v1.8 (Drummond et al., 2012). A birth-death speciation process (Gernhard, 2008) was specified as a tree prior with a death rate parameter sampled from a U(0,1) prior distribution, and a growth rate parameter sampled from a U(0,inf) prior distribution. Rate heterogeneity among lineages was modeled using an uncorrelated lognormal relaxed molecular clock (Drummond et al., 2006) with a mean sampled from an Exp(10) prior distribution. We used a secondary calibration to set the prior on the age of the root using a N(85,8) prior distribution; this parameterization accounts for the uncertainty surrounding the age of the Sapindales (Muellner et al., 2006; Magallón and Castillo, 2009). We used the six Terebinthaceae fossils (see above) to set priors on six nodes: the most recent common ancestor (MRCA) of *Cotinus*, the MRCA of *Loxopterygium*, the MRCA of *Anacardium*, the MRCA of the Protieae, the MRCA of *Commiphora*, and the MRCA of *Bursera* subgenus *Elaphrium*. Because all of these fossils are fragmentary, it is not possible to be certain that any of those fossils possess features that would place them in the crown groups. Therefore, we took a conservative approach and used them as minimum calibrations of the stem groups (Forest, 2009). All these nodes were parameterized with Exponential distributions in which the offset matched the minimum bound set by the fossil age, and the mean was set to be 10% older than this value. Because random starting trees did not satisfy the temporal and topological constraints associated with some fossil calibrations, we used ExaML v1.0.12 (Stamatakis and Aberer, 2013) to estimate a maximum likelihood tree, transformed it into a chronogram using penalized likelihood (Sanderson, 2002; Paradis, 2013), and used it as starting topology in BEAST. The MC2 was run for 6 × 10^7^ generations sampling every 4 × 10^3^ with the first 20% of the samples discarded as burn-in. Convergence to stationarity of the MC2 sampling was determined with time-series plots of the likelihood scores and cumulative split frequencies, and assessing that estimated effective sample sizes for the chronograms and model parameters were at least 100. Post burn-in chronograms were summarized with a majority clade credibility tree (MCCT) using median branch lengths.

We carried out diversification analyses in two ways. First, we used BayesRate (Silvestro et al., 2011) to evaluate whether a single birth-death diversification process for the whole Terebinthaceae, or two birth-death diversification processes, one for Anacardiaceae and one for Burseraceae, better explain the accumulation of lineages through time. For this analysis, we used flat priors, clade-specific taxon sampling proportions (*P*_*Anacardiaceae*_ = 0.21, *P*_*Burseraceae*_ = 0.19), we unlinked rates between clades, and ran the MC2 for 1 × 10^5^ generations, sampling every 1 × 10^2^, and discarding the first 10% as burn-in. For model selection, we used Bayes Factors using the marginal likelihoods calculated using thermodynamic integration. Second, we used BAMM (Rabosky, 2014) to automatically detect shifts in diversification process through time without defining tree partitions *a priori*. For this analysis, we used 1.0 for the Poisson rate prior, the lambda initial prior, and the extinction rate prior. We included a global taxon sampling proportion *P* = 0.20. We ran 1 × 10^7^ generations of MC2, sampling every 1 × 10^3^, and discarding the first 10% as burn-in, with two independent runs to assess convergence.

### Geographic range and climatic niche evolution analyses

To study geographic range evolution through time, we employed maximum-likelihood inference of geographic range evolution using the dispersal, extinction, and cladogenesis (DEC) model (Ree et al., 2005; Ree and Smith, 2008) implemented in Lagrange version 0.1β, and estimated split and ancestral states concurrently. We described the geographic distributions of each Anacardiaceae and Burseraceae taxon following the biogeographic divisions of Good (1974) and Olson and Carlquist (2001) with some modifications. We assigned each species to one or more of the following seven areas: NA: North America (including Central America and the Caribbean); SA: South America; EA: Eurasia (including North Africa/Mediterranean/Arabian Peninsula); SSA: sub-Saharan Africa; MAD: Madagascar, SeA: Southeast Asia (including India); and OC: Oceania (including Papua New Guinea/Tropical Australia/New Caledonia, and Tropical Pacific Islands). We ran a single DEC unconstrained model assuming rates of dispersal/expansion and extinction were uniform across the areas in the model and across the phylogeny. We estimated D, the dispersal/expansion rate across the phylogeny (Ree and Smith, 2008) for each family, by running two more DEC analyses, one for each family.

To study climatic niche evolution through time, we carried out a second DEC analysis. Although DEC was initially designed for modeling geographic range evolution, it provides a sound framework for modeling the evolution of other type of characters (Ree and Smith, 2008), in particular climatic niche. We found two significant benefits of DEC over alternative approaches for modeling the evolution of climatic niche. First, broad climatic niches encompassing two or more unique climatic niches are valid states for single species; many species in nature display broad climatic tolerances. Second, we reasoned that, analogous to geographic range, for an ancestor with a broad climatic niche, climatic adaptation and lineage divergence can result in daughter species inheriting mutually exclusive climatic niches, or one daughter species inheriting one climatic niche, while the other (the remainder of the ancestral lineage) inheriting the ancestral climatic niche (for details and further discussion, see Ree et al., 2005; Ree and Smith, 2008). For this analysis we assigned each species to one or more of the following climatic categories: Temperate (frost-tolerant), Tropical Seasonal Dry Forest/Savannah/Scrubland, and Tropical Moist/Wet Forest. Taxa were assigned to these regions on the basis of the authors' knowledge of the taxa and published sources (Daly et al., 2011; Pell et al., 2011). We ran a single DEC unconstrained model assuming rates of dispersal/expansion and extinction were uniform across the climatic niches in the model and across the phylogeny. We estimated D, the dispersal/expansion rate across the phylogeny (Ree and Smith, 2008), for each family by running two more DEC analyses, one for each family.

## Results

Genbank accession information for all taxa is listed in Table 1. Alignments, analyses and trees generated by the study are posted to Treebase, study number 16073 (www.treebase.org).

### Phylogenetic relationships

**Figure 3:**
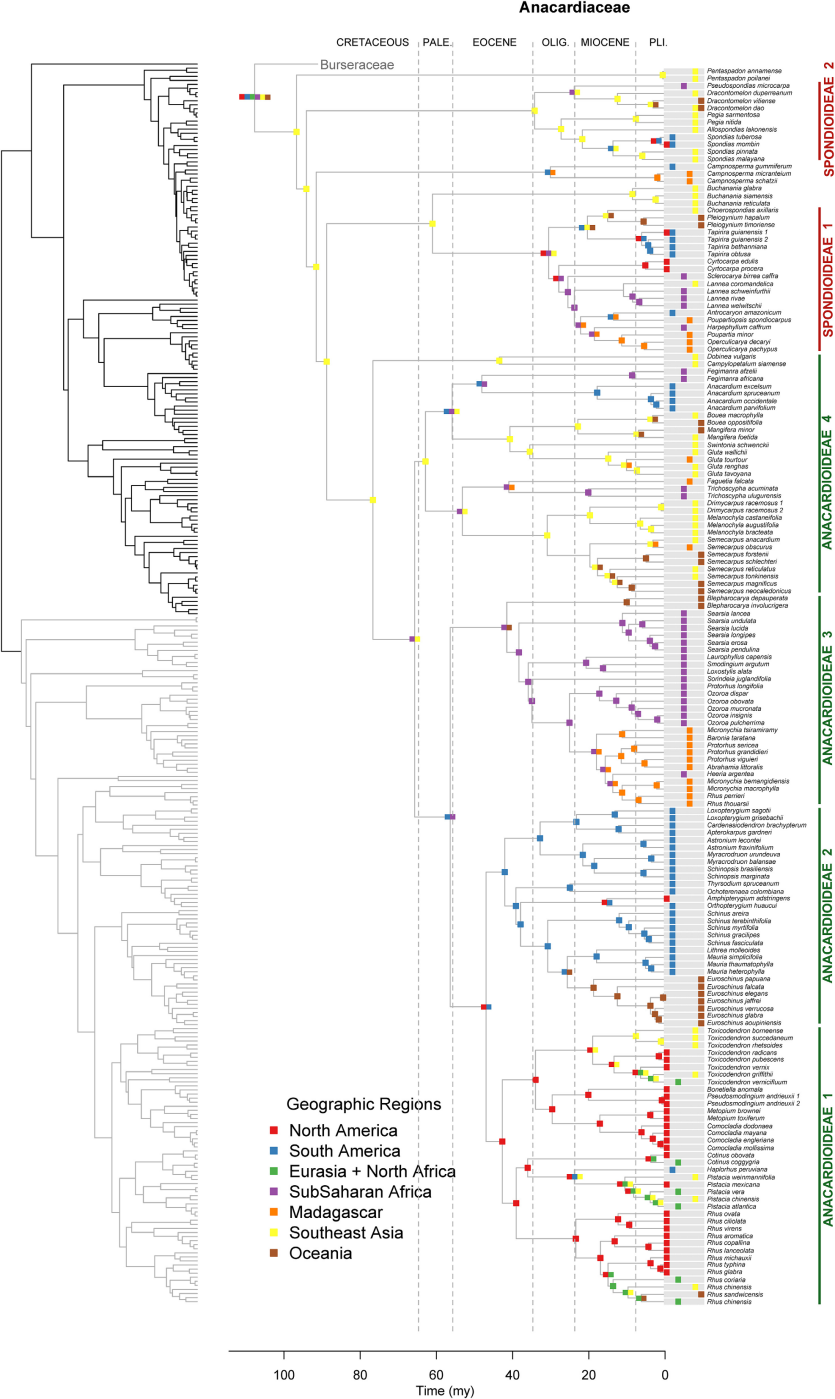
**Maximum likelihood reconstruction of geographic range evolution for Anacardiaceae on the maximum clade credibility tree**. Ancestral geographic ranges are shown at nodes. For each species, the extant geographic range is represented by colored boxes.

Results support the monophyly of Anacardiaceae and Burseraceae individually and their relationship as sister clades (Figure 2). Within Anacardiaceae, phylogenetic results pose challenges to all published subfamilial classifications of the family (Figure 3). The most widely used classification of the family includes five tribes, Anacardieae, Dobineae, Rhoeae, Semecarpeae, Spondiadeae, (Engler, 1881, 1883, 1892; modified by Mitchell and Mori, 1987; and again by Pell, 2004), only two of which are monophyletic as circumscribed, Dobineae (*Campylopetalum* and *Dobinea*) and Semecarpeae (represented here by *Drimycarpus*, *Melanochyla* and *Semecarpus*, but also including *Holigarna* and *Nothopegia*). Pell and Mitchell (Mitchell et al., 2006) proposed the most recent classification, which includes two subfamilies, Anacardioideae and Spondioideae, both of which are shown here to be polyphyletic. Our results do provide some clarity for the position of three genera that have been difficult to place within the evolutionary context of the family: *Buchanania, Campnosperma*, and *Pentaspadon*. Although it is most often treated as a member of tribe/subfamily Anacardieae/Anacardioideae, *Buchanania* is here resolved sister to a clade of taxa traditionally recognized within tribe/subfamily Spondiadeae/Spondioideae. *Campnosperma* and *Pentaspadon* remain challenging but are resolved as sister lineages to much larger clades: *Pentaspadon* is sister to the rest of Anacardiaceae and *Campnosperma* is sister to a clade that includes all of subfamily Anacardioideae included in our sampling (excluding *Pentaspadon* from the Mitchell et al. (2006) circumscription; *Buchanania* + clade of *Dobinea* to *Rhus chinensis* Mill.) and most of Spondioideae (clade containing *Choerospondias* to *Operculicarya*).

**Figure 4:**
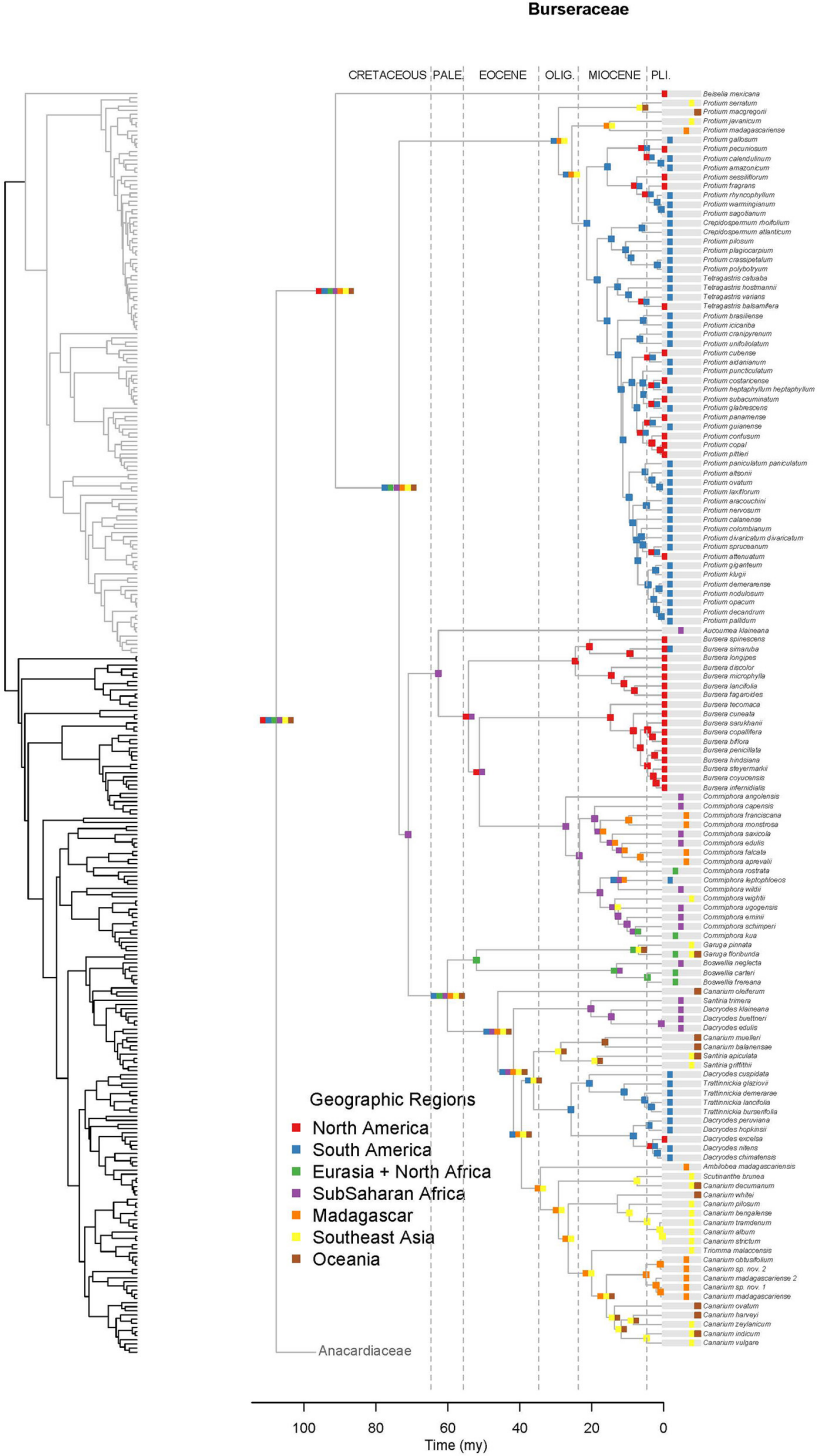
**Maximum likelihood reconstruction of geographic range evolution for the Burseraceae on the maximum clade credibility tree**. Ancestral geographic ranges are shown at nodes. For each species, the extant geographic range is represented by colored boxes.

Within Burseraceae, phylogenetic relationships show the monotypic Mexican species *Beiselia mexicana* sister to a clade containing three well-supported lineages that correspond to the *Protium* alliance or Protieae (sensu Thulin et al., 2008), the *Bursera* alliance or Bursereae (sensu Thulin et al., 2008) and the *Boswellia* + *Canarium* alliance hereafter referred to as Garugeae (sensu Thulin et al., 2008) (Figure 4). Within Protieae, a clade of Southeast Asian and Madagascan species is sister to the species-rich American clade containing the remaining *Protium* species and the genera *Tetragastris* and *Crepidospermum* (see also Fine et al., 2014). Within Bursereae, the monotypic West African *Aucoumea klaineana* is sister to a well-supported clade containing American *Bursera* and predominantly African *Commiphora*. Our study finds marginal support for a paraphyletic *Bursera* with the lineage corresponding to *Bursera* subg. *Bursera* sister to *Commiphora* and *Bursera* subg. *Elaphrium*. More robustly sampled phylogenies of Anacardiaceae and Burseraceae (Pell et al. in prep.; Weeks et al. in prep.) will expand on the brief taxonomic results presented in this publication.

### Timing of diversification and diversification patterns

**Figure 5:**
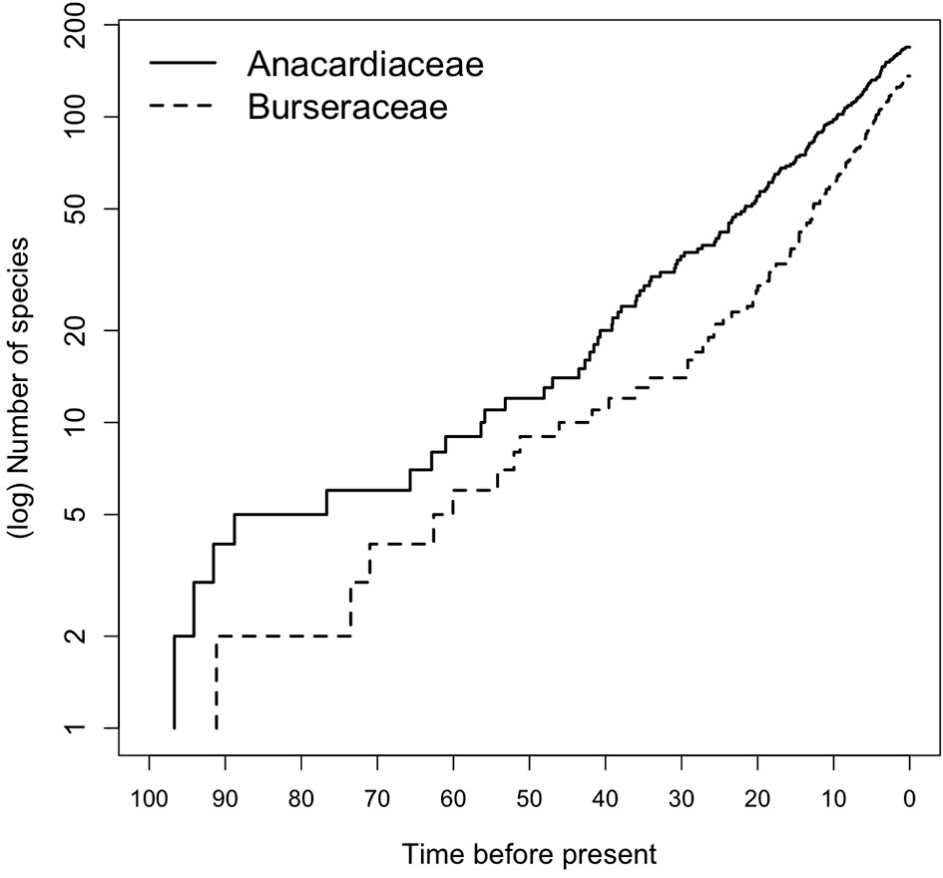
**Lineage through time (LTT) plot for Anacardiaceae and Burseraceae phylogeny**.

Early diversification events in Terebinthaceae including stem and crown divergences of both families occurred in the Early to Late Cretaceous (Figure 2, Supplementary Material Figures S1A,B). The divergence of Terebinthaceae from the other Sapindales lineages and its split into Anacardiaceae and Burseraceae spanned the Albian–Aptian of the Early Cretaceous (116 Ma, 105–127 Ma; mean age, 95% HPD and 108 Ma, 95–121 Ma, respectively). The crown radiations of Anacardiaceae (97 Ma, 83–128 Ma) and Burseraceae (91 Ma, 78–106 Ma) were centered on the Cenomanian of the Late Cretaceous. Lineage through time (LTT) plots show that even though the radiation of Anacardiaceae is slightly older than that of Burseraceae, accumulation of lineages through time has been roughly equivalent in both families (Figure 5). Nevertheless, there is evidence that two, rather than one diversification process has governed the evolution of these families. The highest marginal likelihood was assigned to the model with different Birth-Death diversification processes for each family (*LM* = −1188.75). A Bayes Factor analysis shows very strong evidence in favor of this model against a model with a single Birth-Death diversification process for the whole tree (*BF* = 12). However, the mean net diversification rate for both families is approximately the same (2.4 vs. 2.5). BAMM analysis found strong support for a model with one rate shift, with a posterior probability *p* = 0.74. Bayes Factors show strong evidence in favor of this model vs. a null model with no shifts (*BF* = 574.73). These results suggest a substantial increase in speciation rate in the ancestral lineage leading to the Protieae within Burseraceae (Supplementary Material Figure S2). The posterior probability of a rate shift occurring in the three deepest branches of the Protieae is *p* = 0.91. Bayes Factors indicate overwhelming evidence in favor of a rate shift in the branch leading to the Neotropical Protieae (*BF* = 2004.07).

### Geographic range and climatic niche evolution

Lagrange analyses show there is uncertainty in the geographic ranges and climatic niches for several ancestors (Supplementary Material Figure S3, Tables S1, S2). We define uncertainty as when multiple ancestral states are within two log-likelihood scores. With this in mind, we restrict our description of results and discussion only to the most likely reconstructions (i.e., the one with the highest relative probability).

**Figure 6:**
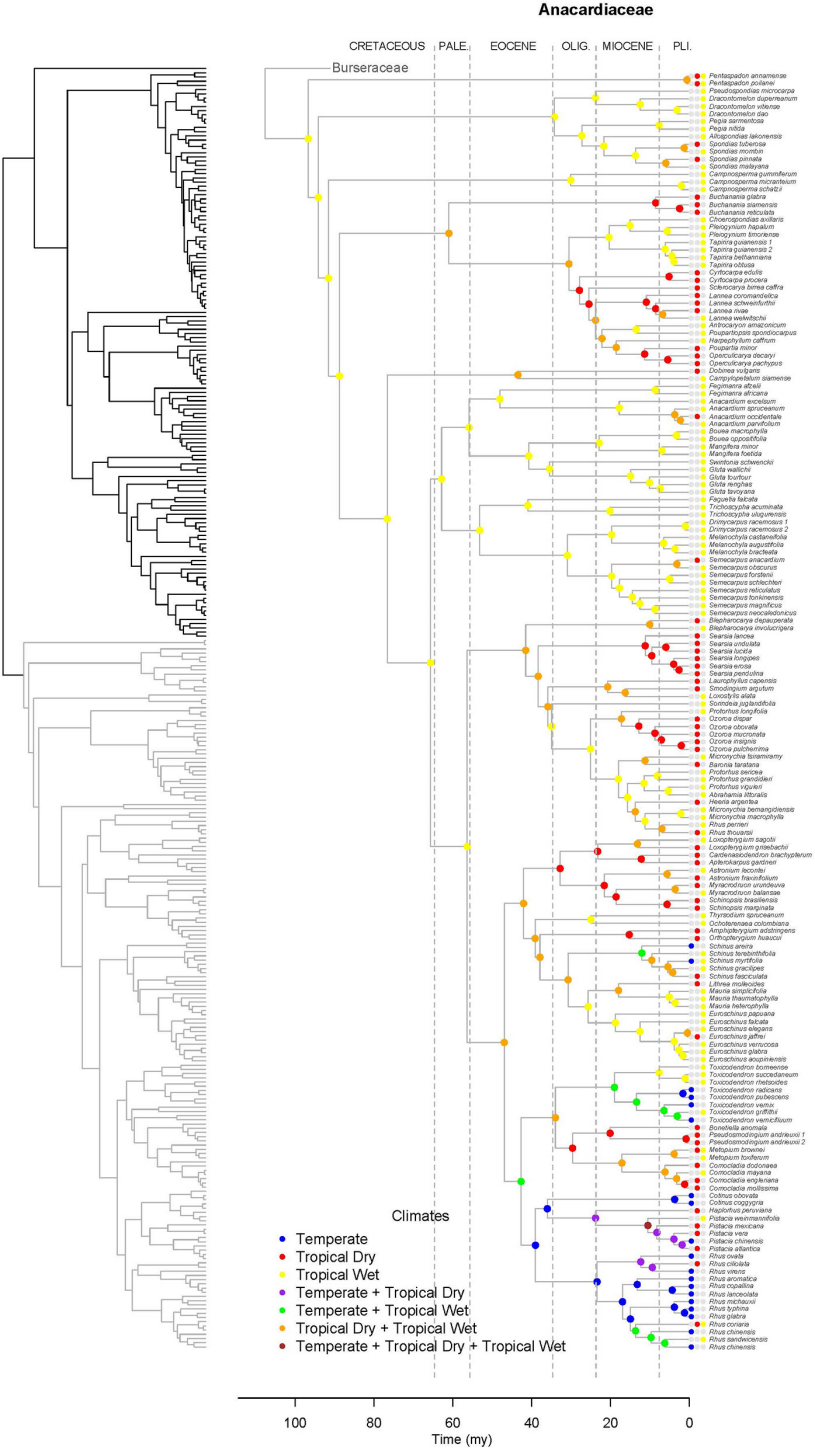
**Maximum likelihood reconstruction of climatic niche evolution for Anacardiaceae on the maximum clade credibility tree**. Ancestral climatic niches are shown at nodes. For each species, the current climatic niche is represented by colored circles.

**Figure 7:**
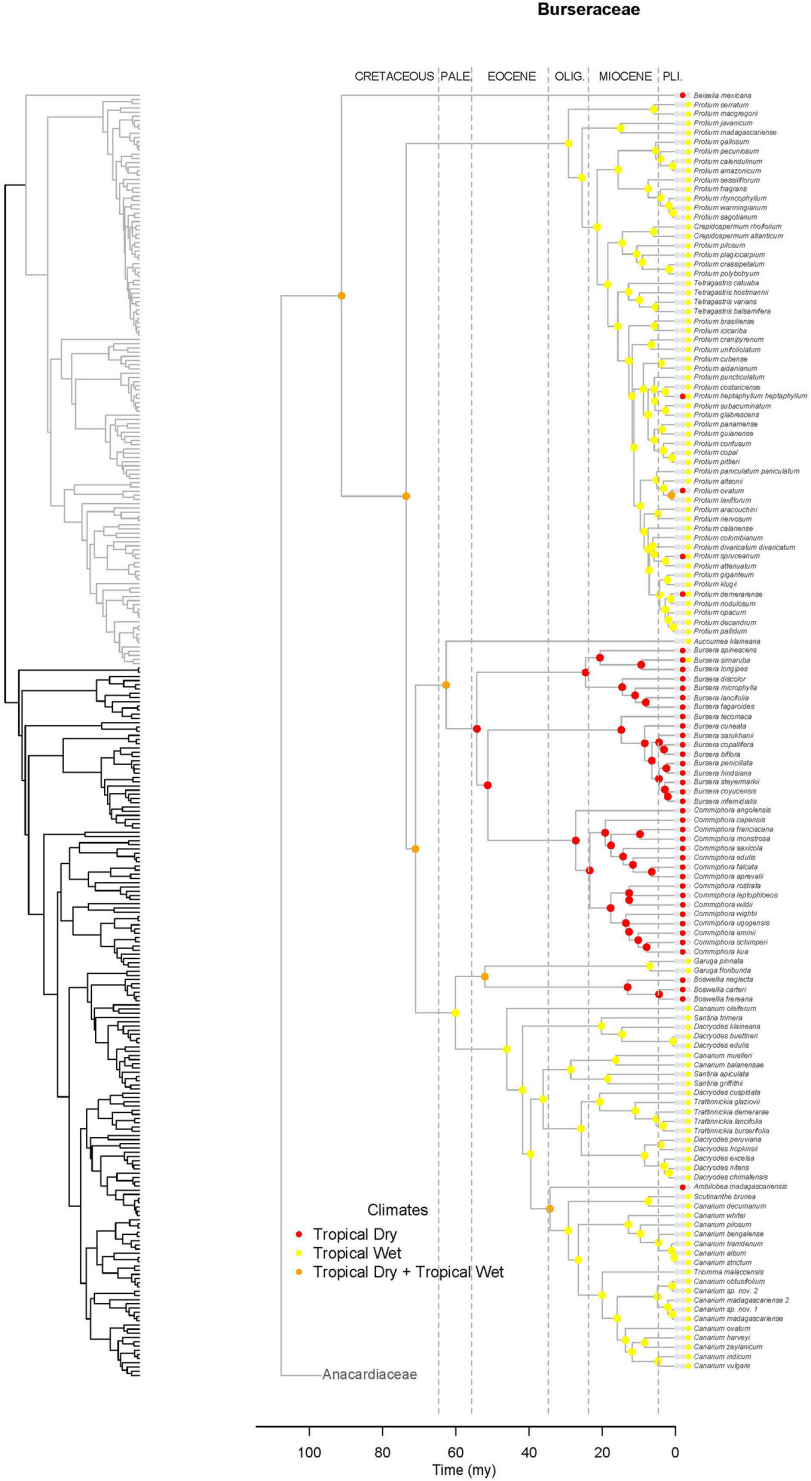
**Maximum likelihood reconstruction of climatic niche evolution for Burseraceae on the maximum clade credibility tree**. Ancestral climatic niches are shown at nodes. For each species, the current climatic niche is represented by colored circle.

Lagrange analysis indicates that the most recent common ancestor of Terebinthaceae had a widespread geographic range and it occurred in wet and dry tropical climatic niches (Figures 6, 7). Speciation within this broad geographic range led to a lineage restricted to tropical wet climates in Southeast Asia (for the most recent common ancestor of Anacardiaceae), and to a lineage inheriting the widespread ancestral geographic range and ancestral climatic niche (for the most recent common ancestor of Burseraceae). Within Anacardiaceae, subsequent speciation events during the Cretaceous occurred within Southeast Asia with a geographic range expansion into the tropical wet forests of sub–Saharan Africa around the Cretaceous–Paleocene boundary. During the Paleocene, geographic range expansion into sub-Saharan Africa led to allopatric divergence, with a daughter species inheriting the Southeast Asian geographic range and its sister species inheriting the sub-Saharan African range and further expanding into South America. The Southeast Asian ancestor later diverged into descendant species that expanded their geographic ranges to sub-Saharan Africa and colonizing South America. Although these geographic range expansions did not involve changes in the ancestral Tropical Wet climatic niche, the most recent common ancestor of *Buchanania* and *Operculicarya* expanded into tropical dry climates in Southeast Asia. During the Eocene, Anacardiaceae dispersed into North America, Oceania, and Madagascar, with some ancestors from tropical wet climatic niches expanding into tropical dry as well as temperate climatic niches. From the Miocene forward, there were multiple ancestral geographic range and climatic niche expansions, including the colonization of Eurasia and diversification in temperate climates. Overall, the predominant pattern in the geographic range and climatic niche evolution of Anacardiaceae is that ancestors with widespread geographic ranges and broad climatic tolerances were common and persisted through multiple speciation events, suggesting that dispersal and evolution of climatic tolerances are common in this family. The overall rate of dispersal/expansion for geographic range evolution was *D* = 0.15, and it was *D* = 0.14 for climatic niche evolution. Transitions between climatic niches were extremely common and included 74 instances of expansions or specializations to new niches (Figure 6).

Unlike Anacardiaceae, Burseraceae did not experience as many geographic range expansions and clearly fewer climatic niche expansions (Figure 7). In general, several ancestors with widespread geographic ranges spanned multiple speciation events. However, multiple ancestors diversified within distinct geographic regions. The overall rate of dispersal/expansion for geographic range evolution was *D* = 0.22. In contrast, few ancestors occurred across wet and dry tropical climates, and these quickly specialized to either tropical wet or tropical dry climates. The overall rate of dispersal/expansion for climatic niche evolution was *D* = 0.05. In total, there were only 11 cladogenic events that coincided with the transition from broad tropical climates to tropical dry or tropical wet climates. The only exception to this pattern is the Garugeae clade in which widespread geographic ranges among ancestors were maintained across multiple cladogenic events. Nevertheless, this clade remained highly specialized to tropical wet climates.

## Discussion

### Origins of terebinthaceae and patterns of diversification in anacardiaceae and burseraceae

The Cretaceous age of the stem and crown lineages of Terebinthaceae (95–127 Ma) coincides with several episodes of continental vicariance that may have contributed to their early widespread distribution and spurred their diversification through allopatric speciation. Biogeographic reconstructions place the common ancestor of extant Anacardiaceae in Southeast Asia and extant Burseraceae in virtually all pantropical locations soon after their divergence. This reconstruction, combined with the Northern Hemisphere distribution of pre-Eocene Terebinthaceae fossils and the earliest diverging extant taxa, points most strongly to an origin of Terebinthaceae in Laurasia rather than Gondwana. In Burseraceae, the oldest fossils attributable to the clade derive from the Paleocene and Eocene of North America and Britain (Daly et al., 2011). Definitively Anacardiaceae fossils appear in the Eocene floras of North America and Europe (e.g., Taylor, 1990; Manchester, 1994; Ramírez and Cevallos-Ferriz, 2002; Meyer, 2003). An intriguing possibility is that the northward incursion of the Atlantic Ocean between North America and Eurasia/proto Southeast Asia during the Upper Cretaceous (Seton et al., 2012) contributed to these early diversification events by interrupting gene flow among the increasingly isolated regions of Laurasia.

After the divergence of the families, the Upper Cretaceous brought crown radiations and the establishment of at least four lineages in Burseraceae and six in Anacardiaceae (Figures 2–4 and Supplementary Material). Timing of the older diversification events within Burseraceae is broadly comparable to that found by previous studies of the family. Similar studies in Anacardiaceae are lacking, with the biogeography of only some of the more recently diversified clades having been evaluated. Our study places the crown radiation of Burseraceae firmly in the Upper Cretaceous, notably older than the Paleocene age estimated by Weeks et al. (2005) and Fine et al. (2014). This discrepancy may be caused by the inclusion of the species sister to all other members of the family, *Beiselia mexicana*, a monotypic genus distributed in western Mexico. It has highly divergent DNA sequences and may inflate older ages as a consequence of its effect on the DNA alignment across Sapindalean taxa.

During the Cretaceous, Anacardiaceae accumulated more lineages than Burseraceae, either through increased speciation or decreased extinction (Figures 2–5). Diversifications of Burseraceae also span the K-T boundary. The stem and crown ages of Burseraceae's Protieae, Bursereae and Garugeae clades closely match the Paleocene ages estimated by studies that sample fewer Sapindalean outgroups (Weeks et al., 2005; De-Nova et al., 2012; Fine et al., 2014). Phylogenetic relationships resolved within these large clades reveal the ancient but relatively rapid establishment of pantropical distributions.

Our analyses of diversification through time indicate that the evolutionary history of Terebinthaceae has been shaped by a mixture of heterogeneous processes. We found strong evidence for an explosive burst in speciation associated with the origin of the Neotropical Protieae. Fine et al. (2014) also found support for a rate shift for this clade using a different modeling approach. Taken together, this suggests that the acceleration in rates during this time interval likely reflects the occurrence of a key innovation or the colonization of a new geographic region and open ecological opportunities. It is noteworthy that similar geographic range expansions did not lead to rate shifts elsewhere in the history of Terebinthaceae [e.g., the clade *Bursera* (Burseraceae) or the clade Anacardioideae 2 (Anacardiaceae)]. Although sampling bias may influence the inference of diversification dynamics, the fast and recent radiation of the Neotropical Protieae (Fine et al., 2014) deeply altered the steady the accumulation of lineages in the history of Terebinthaceae from the Cretaceous to the present. Increased sampling in other species-rich clades such as *Bursera* could inform whether more rate shifts have shaped the evolutionary history of Terebinthaceae.

### Geographic range evolution: have lineages persisted in unique geographic regions or have they dispersed to new geographic regions (i.e., how common is dispersal)?

Long-distance dispersal features prominently in the biogeographic history of Terebinthaceae. Although dispersal rates estimated with Lagrange may not be precise (Ree and Smith, 2008), our results suggest that dispersal rates for both Anacardiaceae and Burseraceae are relatively high and similar (*D*_*Anacardiaceae*_ = 0.15, *D_*Burseraceae*_* = 0.22). While frequency of long-distance dispersal in plants is not necessarily correlated to dispersal syndrome (Higgins et al., 2003), seeds of the majority of Burseraceae and Anacardiaceae taxa are dispersed by animals (esp. birds, bats, terrestrial mammals; Daly et al., 2011; Pell et al., 2011). Some members of both families are wind-dispersed and a few Anacardiaceae species are water dispersed. A closer examination of Terebinthaceae evolution reveals cases in which repeated short-distance dispersals or extreme long-distance dispersal must be invoked. In Burseraceae, within Garugeae, separation of Southeast Asian and African taxa occurs (*Boswellia*, *Garuga*; 52 Ma, 33–68 Ma) along with a separation of a New Caledonian endemic taxon, *Canarium oleiferum*, from the remaining pantropical Garugeae clade (46 Ma, 36–59 Ma). In Anacardiaceae, sister lineages from South America and sub-Saharan Africa diverge (*Anacardium, Fegimanra;* 48 Ma, 47–51 Ma), African and Oceanian taxa split (*Blepharocarya*, rest of Anacardioideae ‘2 Ma, 30–56 Ma), Madagascan and African taxa diverge (*Faguetia, Trichoscypha*; 41 Ma, 25–58 Ma), and South and North American lineages diverge (Anacardioideae 1, 2; 47 Ma, 39–57 Ma). The similar timing of these biogeographic expansions suggests relatively rapid dispersal among continents followed by radiation within continental regions but does not indicate shared routes. For instance, Fine et al. (2014) posits that the predominantly South American Protieae derived from an ancestor that dispersed across the Atlantic Ocean from North America or Africa, whereas Weeks and Simpson (2007) suggest the ancestor of Paleotropical *Commiphora* migrated from the Americas to the Old World across the North Atlantic boreotropical land-bridge.

Closer examination of the biogeographical reconstructions of these two families reveal repeated instances of lineage divergences associated with two different regions and/or continents. These splits occurred throughout the past 100 million years, including some very recent events. While some of these divergences may have been due to vicariance, we believe the great majority of them have been due to long-distance dispersal events. Recent reviews of the biogeographic history tropical woody plant lineages have emphasized the importance of long-distance dispersal (Lavin et al., 2004; Pennington and Dick, 2004; Renner, 2004). Our results support this view, and we conclude that both Anacardiaceae and Burseraceae have moved easily across oceans.

### Climatic niche evolution: have lineages retained distinct climatic niches or have they evolved climatic tolerances (i.e., how common is “niche expansion”)?

Burseraceae are characterized by a low degree of climatic niche evolution. There are no frost-tolerant species, i.e., all Burseraceae are restricted to tropical and subtropical latitudes. Although Burseraceae are common and even dominant elements in seasonally-dry tropical forests and xeric scrublands as well as moist/wet rain forests across the tropics, switches between wet and dry climates are relatively rare in the family (Figure 7). For example, *Commiphora* and *Bursera*, which dominate some seasonally-dry regions of sub-Saharan Africa and Mesoamerica respectively, share a common ancestor that almost certainly was a dry forest specialist. Both dry-forest and wet-forest lineages are ancient in Burseraceae, and both climatic niches have included Burseraceae taxa since before the Paleocene.

Unlike Burseraceae, transitions among climatic niches are extremely common in Anacardiaceae. Our estimate of the overall rate of dispersal/expansion for Anacardiaceae is at least twice the rate in Burseraceae (*D*_*Anacardiaceae*_ = 0.14, *D*_*Burseraceae*_ = 0.05). In the Lagrange analysis (Figure 6), the most recent common ancestor of all Anacardiaceae and all deep nodes within the family are hypothesized to be wet forest taxa until the first expansion into dry climates during the Paleocene and then many more during the Eocene. Colonization of the temperate zone occurred early in Anacardiaceae evolution, likely during the Eocene. There have been at least 74 climate transitions (expansions and specializations) across all climatic niches examined here. Interestingly, although transitions between wet and dry climates are very common in Anacardiaceae, expansion into the temperate climate appears to have occurred in only one clade (although may have been lost and re-evolved in the same clade several times). This clade includes a broad selection of genera primarily in the Americas with a scattering of taxa from Europe, Asia, and the Pacific. Clades within this lineage in which frost tolerance has evolved are *Cotinus*, *Pistacia*, *Rhus* s.s., *Schinus*, and *Toxicodendron*. Within this clade, there have been multiple transitions among temperate and wet and dry tropical climates.

It is clear that climatic niche evolution is not integral to explaining the high species diversity of Burseraceae, as close relatives almost always occur in the same climatic niche. However, for Anacardiaceae, it is tempting to make a connection between climatic niche evolution and diversification. For several clades, sister species (or sister clades) share the same geographic region but inhabit different climatic niches, suggesting that specialization to dry or wet (or temperate vs. tropical) could arise after a widespread taxon lived in more than one climate. Tradeoffs involved in drought or frost tolerance are likely involved in such specialization. For example, Pittermann et al. (2012) showed that adaptation to dry biomes by Cupressaceae trees involves cavitation resistant xylem which results in reduced photosynthetic rates causing low growth rates which presumably prevents dry-adapted lineages from competing successfully in wet biomes.

Wind dispersal occurs in both families but to a greater degree of frequency and complexity in Anacardiaceae. Burseraceae have five wind-dispersed genera (*Aucoumea*, *Ambilobea*, *Beiselia, Boswellia* and *Triomma*; ca. 24 species) in all of which wings are obtained via conplanation of the pyrene, whereas Anacardiaceae have 23 genera, including ca. 75 species, in which a wide variety of mechanisms have evolved to facilitate wind dispersal. These include, for example, wings developed from petals, sepals, pericarp, subtending bracts, and whole inflorescences. Wind dispersal is more common in tropical dry forests than in tropical wet forests (Gentry, 1982, 1991), and thus perhaps affords an evolutionary advantage in this habitat. Fruit structure in Anacardiaceae may be more plastic than in Burseraceae, suggested by the fruit diversity in the family and the multiple times wind dispersal has evolved through the modification of different structures. Anacardiaceae, after being dispersed to a dry habitat, may have evolved more advantageous wind dispersed fruits quickly *in situ*, or they may have evolved wind-dispersed fruits in wet habitats then dispersed to dry habitats. Physiological responses to environment may also play a role in the ability of Anacardiaceae to change habitats. In some wet to dry switching lineages, like *Astronium*, the moist forest species occur in areas that often have a briefly drier period during which the species may lose their leaves. Ancestors of these lineages may have also had periodic deciduousness, possibly pre-adapting them to more seasonal forests with longer, more extreme dry periods. Other Anacardiaceae lineages include examples of morphological plasticity with respect to leaf morphology. For example in the genera *Loxopterygium* and *Astronium* leaves of most wet-habitat taxa are mostly entire margined, while leaves of most dry-habitat taxa have toothed margins.

In contrast to Anacardiaceae, it appears that the great majority of diversification occurs *within* climatic niches for Burseraceae. There are many mechanisms that could yield this pattern. First, if a lineage has frequent dispersal to the same climatic niche in different geographic regions, allopatric speciation could occur, and then re-dispersal back to the original region could inflate sympatric species totals. Second, habitat specialization to other habitats within climatic niches or niche partitioning along other niche axes within habitats can increase the numbers of species of a lineage within a climatic niche. For example, edaphic specialization to different soil types has been implicated in the diversification of the Protieae (Fine et al., 2005, 2014). Finally, escape from natural enemies through effective chemical defenses may promote species radiations within biomes (Ehrlich and Raven, 1964). Becerra (2007) showed that the terpene defenses of *Bursera* were more divergent than expected by co-occurring species within regions, and she suggested that coevolutionary interactions between Burseraceae-feeding beetles promoted chemical divergence and speciation in this group. Other Burseraceae lineages such as the Protieae have also been shown to express a wide diversity of terpenes and other non-volatile antiherbivore defenses Fine et al., 2006, 2014; Zapata and Fine, 2013.

## Conclusion

We found that a densely sampled, comprehensive geographic sample of Anacardiaceae and Burseraceae taxa has yielded a highly supported phylogenetic reconstruction that supports current taxonomic concepts of both families. Moreover, our fossil-calibrated chronogram and biogeographic analyses give results that are broadly congruent with the fossil record. We conclude that the most common ancestor to these families was widespread and likely originated in Northern Hemisphere during the Cretaceous. Continental vicariance between hemispheres may have spurred initial divergence into Burseraceae and Anacardiaceae and indeed the two families followed different evolutionary trajectories since their split, with Anacardiaceae steadily accumulating lineages since the late Cretaceous–Paleocene while the majority of Burseraceae's diversification has occurred much more recently, with Miocene radiations of the Protieae and Bursereae. Both families have relied on effective wind and animal dispersal to achieve pantropical distributions with multiple intercontinental colonization events inferred for both families throughout the past 100 million years. Anacardiaceae have shifted climatic niches frequently during this time, including colonization of the temperate biomes, while Burseraceae have experienced very few shifts between tropical dry and tropical wet climates, with no temperate zone adaptation. Thus, in the context of the question of whether is it easier for these plant lineages to move or to evolve, we conclude that both Anacardiaceae and Burseraceae move easily, but Anacardiaceae have a much greater capacity to adapt to new climate regimes than Burseraceae and this is one of the most striking features of their evolutionary history.

### Conflict of interest statement

The authors declare that the research was conducted in the absence of any commercial or financial relationships that could be construed as a potential conflict of interest.
