# Supplementary material for: To move or to evolve: contrasting patterns of intercontinental connectivity and climatic niche evolution in “Terebinthaceae” (Anacardiaceae and Burseraceae)
Source: Front Genet. 2014 Nov 28;5:409. doi: 10.3389/fgene.2014.00409 (PMC4247111; doi:10.3389/fgene.2014.00409)
Supplement: Supplementary file 1 [file DataSheet1.PDF]

Figure S1. A. Mean age of nodes as estimated by Beast analysis.

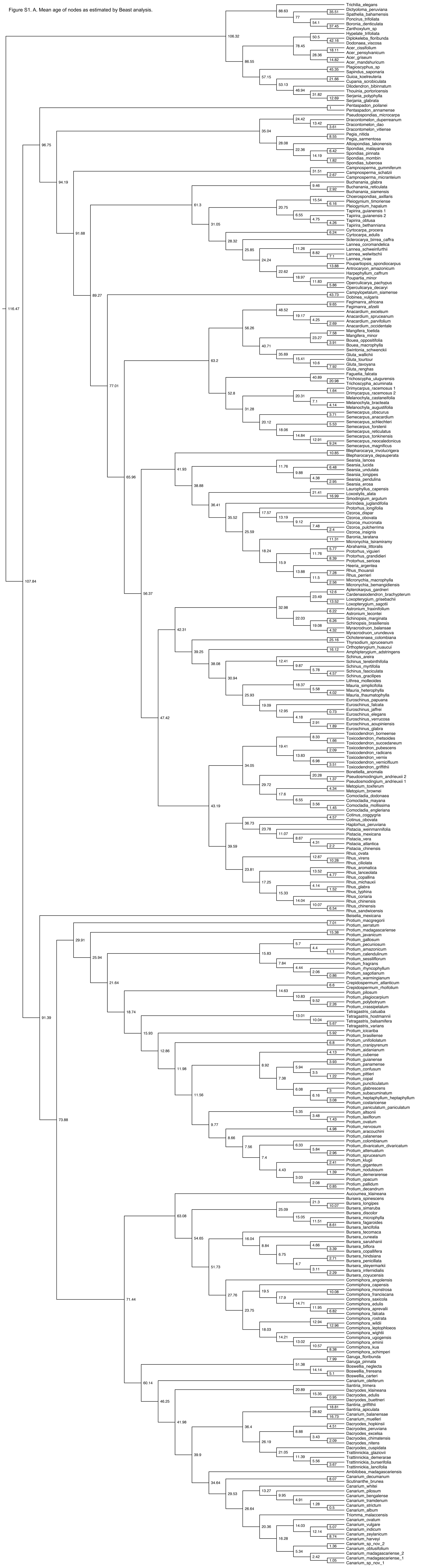

Figure S1. B. 95% highest posterior distribution of node ages (Ma) as estimated by Beast.

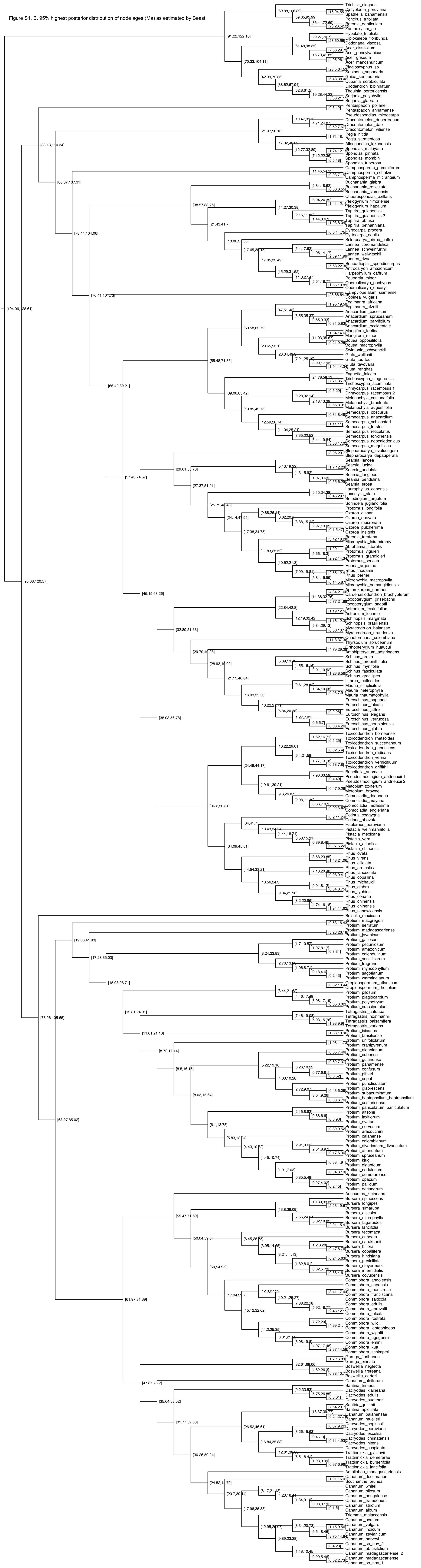

Figure S2. a) Phylorate plot of the first three distinct rate shift configurations that together account for 90% of the probability of the data. b) Maximum a posteriori (MAP) probability rate shift configuration. The node indicates the rate shift happened at the base of the Neotropical Protieae. c) Phylogeny with branch lengths scaled by Bayes Factor evidence for a rate shift. Number on branches correspond to Bayes Factor. The branch with the highest Bayes Factor corresponds to the branch subtending the Neotropical Protieae. For details on Bayes Factor analysis and rate shift configurations, see Rabosky 2014.

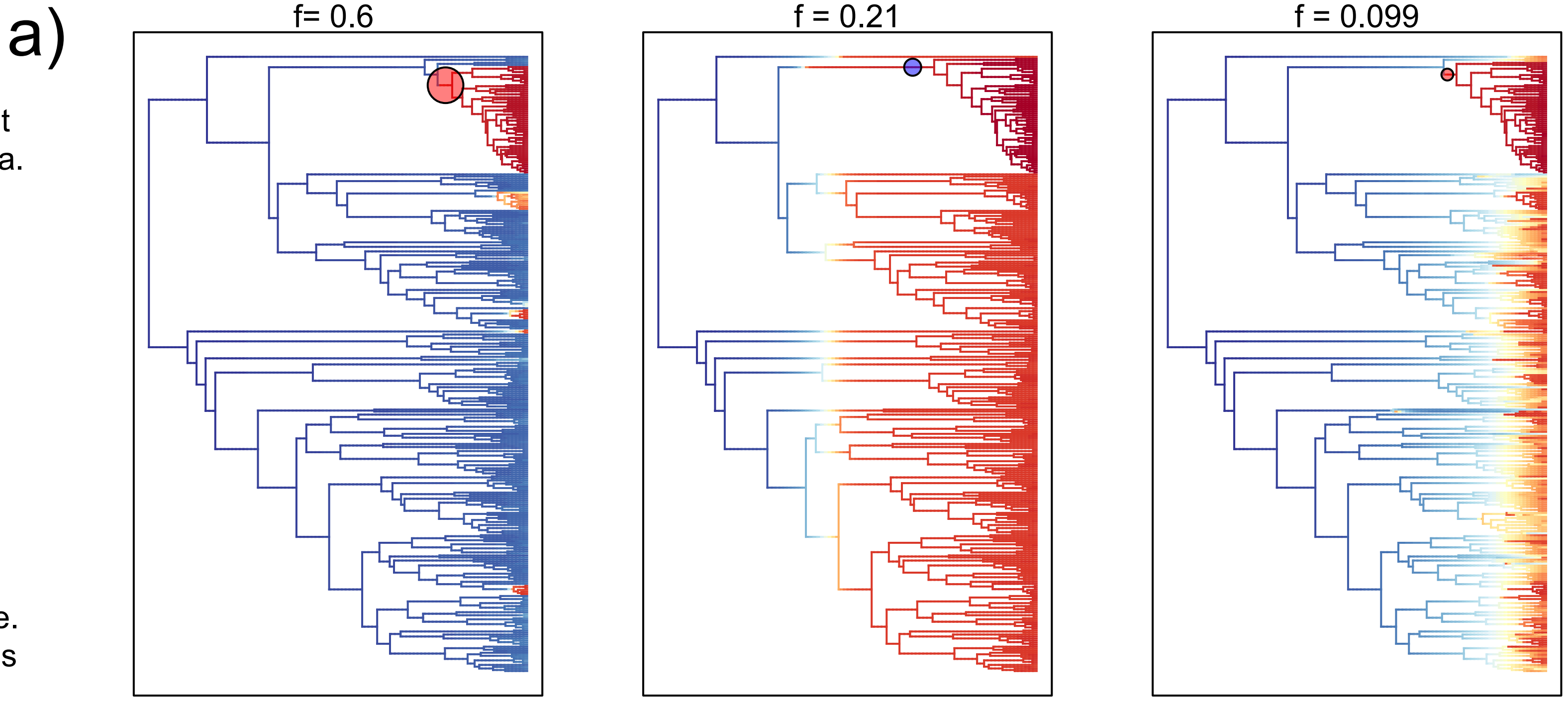

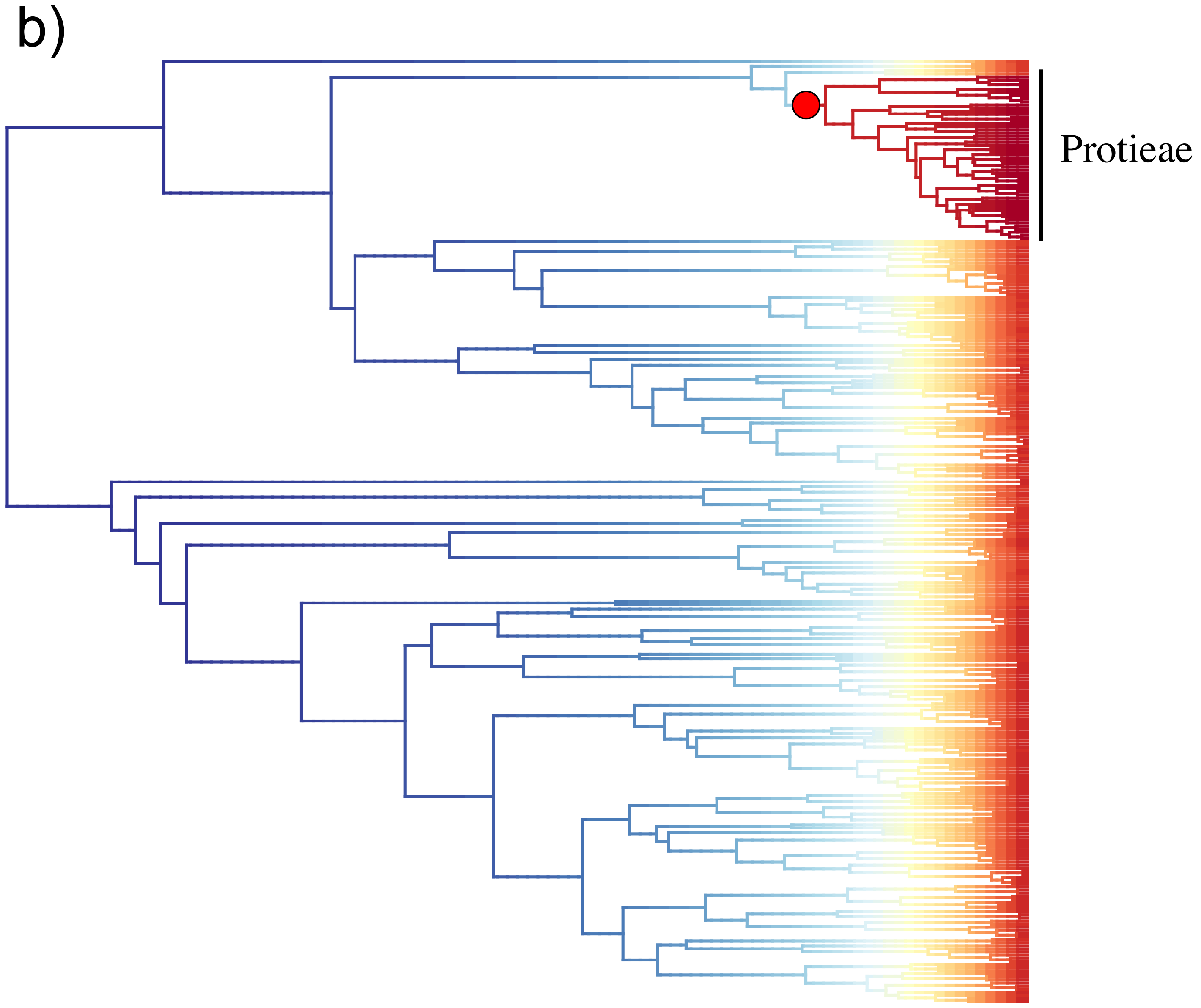

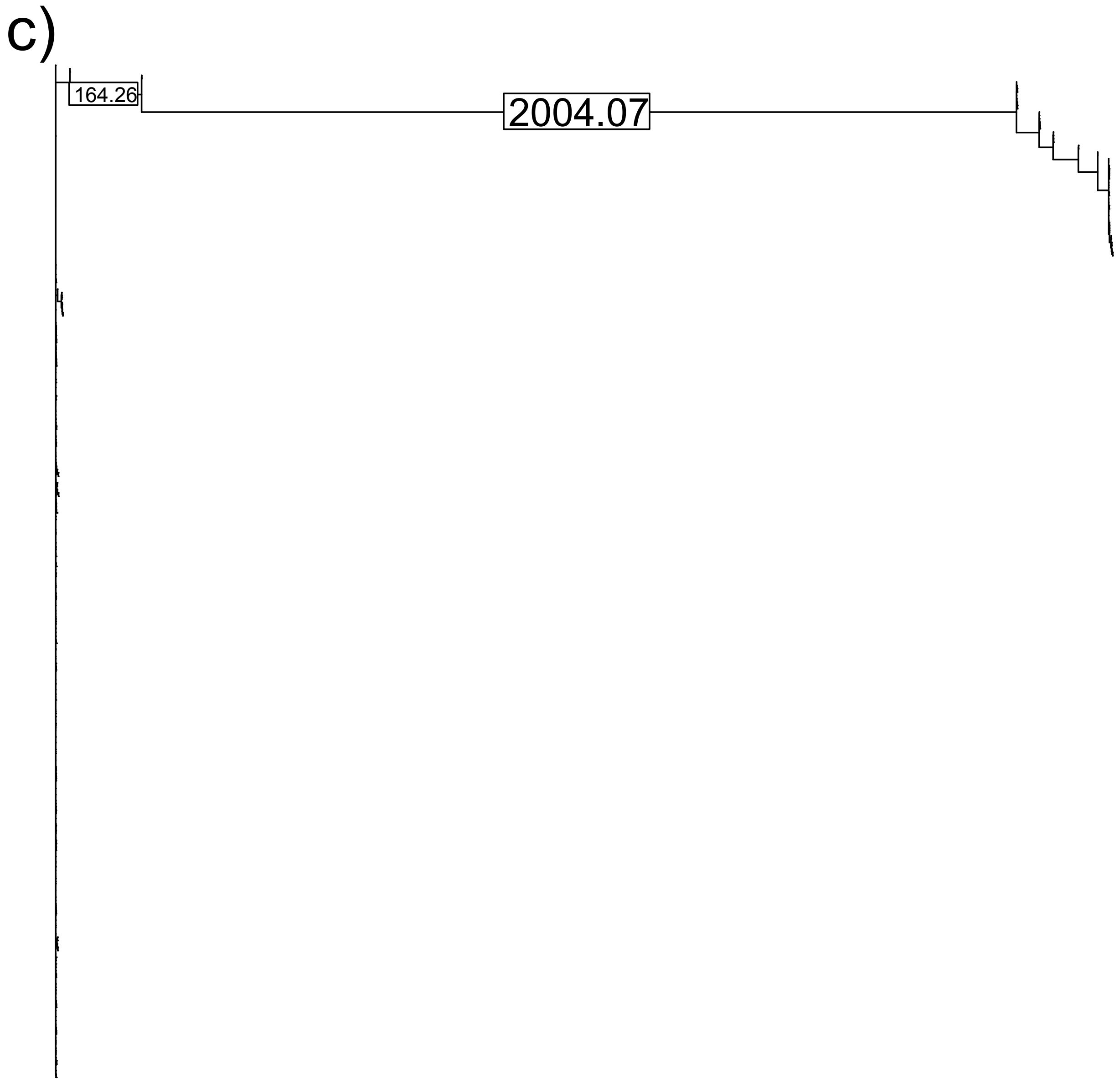

Figure S3. Node numbers for Lagrange analyses.

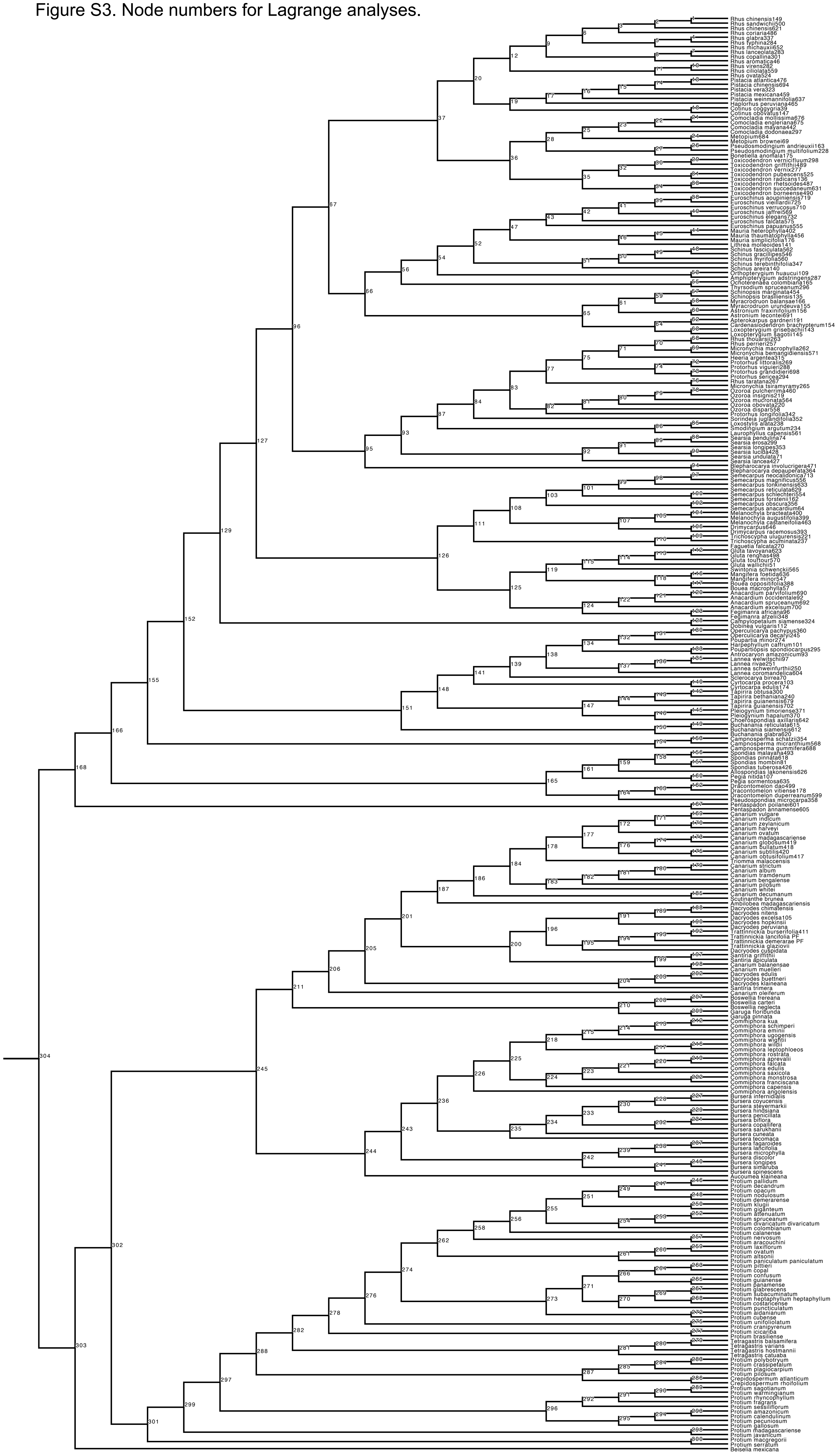

Table S1. Alternative ancestral geographic range reconstructions. P: probability, L: likelihood. Areas: EU+NA (Eurasia, North Africa, Mediterranean, Arabian Peninsula), NA: North America (including Central America and Caribbean), SA (South America), SSA (subSaharan Africa), MAD (Madagascar), SeA (Southeast Asia), OC (Oceania, PNG, Tropical Australia, New Caledonia, Tropical Pacific Islands)

Node	Ancestral State	P	-LOG(L)
1	EU+NA_OC	0.98	475.251
2	EU+NA_SeAS_OC	0.61	475.728
3	EU+NA	0.56	475.809
	EU+NA_SeAS	0.19	476.908
	EU+NA_SeAS_OC	0.14	477.197
	EU+NA_OC	0.09	477.589
4	NA	1	475.231
5	NA	1	475.231
6	NA_EU+NA	0.61	475.72
	NA_EU+NA_SeAS	0.14	477.215
	NA_EU+NA_SeAS_OC	0.1	477.534
7	NA	1	475.231
8	NA	1	475.231
9	NA	0.44	476.059
	NA_EU+NA	0.3	476.43
	NA_EU+NA_SeAS	0.07	477.822
	NA_EU+NA_SeAS_OC	0.06	478.029
10	NA	1	475.231
11	NA	1	475.231
12	NA	0.74	475.529
	NA_EU+NA	0.12	477.367
13	EU+NA_SeAS	1	475.233
14	EU+NA_SeAS	0.96	475.273
15	NA_EU+NA_SeAS	0.75	475.517
	NA_SeAS	0.19	476.877
16	NA_EU+NA_SeAS	0.69	475.607
	NA_SeAS	0.29	476.461
17	NA_SA_SeAS	0.29	476.474
	NA_SA	0.27	476.546
	NA_SA_EU+NA_SeAS	0.22	476.726
	NA_SA_EU+NA	0.14	477.192
18	NA_EU+NA	1	475.234
19	NA	0.58	475.767
	NA_SA	0.15	477.159
20	NA	0.61	475.718
	NA_SA	0.14	477.195
21	NA	1	475.231
22	NA	1	475.231
23	NA	1	475.231
24	NA	1	475.231
25	NA	1	475.231
26	NA	1	475.231
27	NA	1	475.231
28	NA	1	475.231
29	EU+NA_SeAS	0.99	475.236
30	NA_EU+NA_SeAS	0.78	475.483
	NA_SeAS	0.2	476.862
31	NA	1	475.231
32	NA_SeAS	0.53	475.858
	NA_EU+NA_SeAS	0.37	476.221
33	SeAS	1	475.231
34	SeAS	1	475.232
35	NA_SeAS	0.73	475.544
	NA_EU+NA_SeAS	0.25	476.633
36	NA	0.68	475.61
	NA_SeAS	0.25	476.6
37	NA	0.44	476.044
	NA_SeAS	0.2	476.822
	NA_SA	0.15	477.144
	NA_SA_SeAS	0.07	477.913
38	OC	1	475.231
39	OC	1	475.231
40	OC	1	475.231
41	OC	1	475.231
42	OC	1	475.234
43	OC	0.98	475.253
44	SA	1	475.231

Node	Ancestral State	P	-LOG(L)
45	SA	1	475.231
46	SA	1	475.234
47	SA_OC	0.97	475.266
48	SA	1	475.231
49	SA	1	475.231
50	SA	1	475.231
51	SA	1	475.231
52	SA	0.81	475.445
	SA_OC	0.19	476.881
53	NA_SA	0.98	475.256
54	SA	0.9	475.331
55	SA	1	475.232
56	SA	0.92	475.319
57	SA	1	475.231
58	SA	1	475.231
59	SA	1	475.231
60	SA	1	475.231
61	SA	1	475.231
62	SA	1	475.231
63	SA	1	475.231
64	SA	1	475.231
65	SA	1	475.232
66	SA	0.92	475.318
67	NA_SA	0.47	475.977
	NA_SA_SeAS	0.21	476.778
	SA	0.09	477.691
	SA_SeAS	0.07	477.936
68	MAD	1	475.231
69	MAD	1	475.231
70	MAD	1	475.233
71	SSA_MAD	0.99	475.237
72	MAD	1	475.231
73	MAD	1	475.231
74	MAD	1	475.231
75	SSA_MAD	0.97	475.266
76	MAD	1	475.231
77	SSA_MAD	0.96	475.271
78	SSA	1	475.231
79	SSA	1	475.231
80	SSA	1	475.231
81	SSA	1	475.231
82	SSA	1	475.231
83	SSA	0.78	475.474
	SSA_MAD	0.22	476.765
84	SSA	0.99	475.239
85	SSA	1	475.231
86	SSA	1	475.232
87	SSA	0.99	475.237
88	SSA	1	475.231
89	SSA	1	475.231
90	SSA	1	475.231
91	SSA	1	475.231
92	SSA	1	475.232
93	SSA	0.99	475.246
94	OC	0.98	475.252
95	SSA_OC	0.85	475.39
96	SA_SSA	0.13	477.28
	NA_SA_SSA	0.1	477.53
	SA_SSA_OC	0.08	477.754
	NA_SSA	0.08	477.773
	SA_OC	0.06	478.103
	NA_SA_OC	0.05	478.175
	NA_SSA_OC	0.05	478.186
	SA_SSA_SeAS	0.04	478.354
	NA_SA_SSA_SeAS	0.04	478.456
	SSA_SeAS	0.04	478.547
	SA_SeAS_OC	0.04	478.554
	SeAS_OC	0.03	478.667
	NA_SA_SeAS_OC	0.03	478.675
	NA_SSA_SeAS	0.03	478.858
	SSA_SeAS_OC	0.02	479.044
	NA_SeAS_OC	0.02	479.059
	SA	0.02	479.084

Node	Ancestral State	P	-LOG(L)
	NA_OC	0.02	479.095
97	OC	1	475.233
98	SeAS_OC	1	475.233
99	SeAS_OC	0.97	475.257
100	OC	1	475.232
101	SeAS_OC	0.93	475.307
102	MAD_SeAS	1	475.233
103	SeAS_OC	0.45	476.04
	SeAS	0.33	476.327
	MAD_SeAS	0.12	477.374
	MAD_SeAS_OC	0.1	477.555
104	SeAS	1	475.231
105	SeAS	1	475.231
106	SeAS	1	475.231
107	SeAS	1	475.232
108	SeAS	0.69	475.602
	SeAS_OC	0.14	477.195
	MAD_SeAS	0.12	477.357
109	SSA	0.97	475.263
110	SSA_MAD	0.75	475.518
111	SSA_SeAS	0.3	476.432
	MAD_SeAS	0.25	476.605
	SSA_MAD_SeAS	0.2	476.859
	SeAS	0.12	477.335
112	SeAS	1	475.233
113	MAD_SeAS	0.97	475.257
114	SeAS	0.6	475.743
	MAD_SeAS	0.4	476.15
115	SeAS	0.97	475.259
116	SeAS_OC	0.99	475.238
117	SeAS_OC	1	475.233
118	SeAS	0.58	475.774
	SeAS_OC	0.41	476.123
119	SeAS	0.91	475.325
120	SA	1	475.231
121	SA	1	475.232
122	SA	0.96	475.269
123	SSA	0.98	475.252
124	SA_SSA	0.69	475.602
125	SA_SSA_SeAS	0.31	476.402
	SSA_SeAS	0.28	476.517
	SA_SeAS	0.24	476.67
	SeAS	0.13	477.267
126	SeAS	0.4	476.136
	SSA_SeAS	0.26	476.577
	SA_SeAS	0.12	477.346
	SA_SSA_SeAS	0.09	477.688
127	SSA_SeAS	0.26	476.566
	SeAS	0.19	476.902
	SA_SeAS	0.15	477.106
	SA_SSA_SeAS	0.09	477.63
	SeAS_OC	0.05	478.329
128	SeAS	1	475.234
129	SeAS	0.76	475.502
130	MAD	1	475.231
131	MAD	1	475.234
132	SSA_MAD	0.98	475.25
133	SA_MAD	0.95	475.279
134	SSA_MAD	0.87	475.369
135	SSA	1	475.231
136	SSA	1	475.233
137	SSA_SeAS	0.98	475.249
138	SSA	0.49	475.943
	SSA_MAD	0.24	476.668
	SSA_SeAS	0.13	477.273
	SSA_MAD_SeAS	0.11	477.48
139	SSA	0.58	475.769
	SSA_MAD	0.15	477.113
	SSA_SeAS	0.13	477.267
	SSA_MAD_SeAS	0.1	477.568
140	NA	0.99	475.24
141	NA_SSA	0.55	475.826
	NA_SSA_SeAS	0.11	477.426

Node	Ancestral State	P	-LOG(L)
	NA_SSA_MAD	0.11	477.438
	NA_SSA_MAD_SeAS	0.08	477.73
142	SA	1	475.231
143	SA	1	475.231
144	NA_SA	0.58	475.767
	SA	0.41	476.12
145	OC	0.99	475.237
146	SeAS_OC	0.96	475.269
147	SA_SeAS_OC	0.4	476.152
	NA_SeAS_OC	0.24	476.672
	SA_SeAS	0.12	477.317
	NA_SA_SeAS	0.09	477.621
	NA_SeAS	0.07	477.873
148	NA_SSA_SeAS	0.18	476.93
	NA_SeAS	0.13	477.237
	NA_SSA_MAD_SeAS	0.1	477.496
	NA_SeAS_OC	0.1	477.522
	SA_SSA_SeAS_OC	0.07	477.853
	NA_SA_SeAS	0.06	478.072
	NA_SA_SeAS_OC	0.06	478.125
	SA_SSA_SeAS	0.03	478.646
	NA_SSA_SeAS_OC	0.03	478.847
149	SeAS	1	475.231
150	SeAS	1	475.232
151	SeAS	0.66	475.64
152	SeAS	0.9	475.341
153	MAD	0.99	475.237
154	SA_MAD	0.61	475.732
	SA_MAD_SeAS	0.27	476.555
155	SeAS	0.7	475.591
	SA_SeAS	0.16	477.091
	MAD_SeAS	0.12	477.374
156	SeAS	1	475.232
157	NA_SA	0.8	475.456
	SA	0.2	476.85
158	SA_SeAS	0.48	475.969
	NA_SeAS	0.29	476.481
	NA_SA_SeAS	0.21	476.812
159	SeAS	0.84	475.402
160	SeAS	1	475.231
161	SeAS	0.95	475.277
162	SeAS_OC	0.98	475.247
163	SeAS	0.62	475.705
	SeAS_OC	0.37	476.219
164	SSA_SeAS	0.7	475.588
	SSA_SeAS_OC	0.24	476.665
165	SeAS	0.49	475.947
	SSA_SeAS	0.34	476.324
	SSA_SeAS_OC	0.11	477.483
166	SeAS	0.89	475.346
167	SeAS	1	475.231
168	SeAS	0.93	475.305
169	SeAS	0.57	475.8
	SeAS_OC	0.43	476.066
170	SeAS_OC	0.99	475.241
171	SeAS_OC	0.93	475.307
172	SeAS_OC	0.93	475.302
173	MAD	1	475.231
174	MAD	1	475.231
175	MAD	1	475.231
176	MAD	1	475.233
177	MAD_SeAS_OC	0.76	475.505
	MAD_SeAS	0.16	477.066
178	MAD_SeAS_OC	0.45	476.021
	MAD_SeAS	0.24	476.646
	SeAS	0.21	476.795
	SeAS_OC	0.09	477.617
179	SeAS	1	475.231
180	SeAS	1	475.231
181	SeAS	1	475.231
182	SeAS	1	475.233
183	SeAS_OC	0.98	475.252
184	MAD_SeAS	0.28	476.516

Node	Ancestral State	P	-LOG(L)
	SeAS	0.28	476.521
	MAD_SeAS_OC	0.24	476.657
	SeAS_OC	0.1	477.494
	MAD_OC	0.05	478.168
	OC	0.05	478.249
185	SeAS	0.53	475.86
	SeAS_OC	0.47	475.996
186	MAD_SeAS	0.31	476.4
	SeAS	0.26	476.569
	MAD_SeAS_OC	0.18	476.934
	SeAS_OC	0.12	477.392
	MAD_OC	0.07	477.909
	OC	0.06	478.082
187	MAD_SeAS	0.35	476.293
	MAD	0.26	476.573
	MAD_OC	0.16	477.083
	MAD_SeAS_OC	0.16	477.089
	SeAS	0.05	478.241
188	SA	1	475.231
189	NA_SA	1	475.234
190	SA	1	475.231
191	SA	0.58	475.768
	NA_SA	0.41	476.117
192	SA	1	475.231
193	SA	1	475.231
194	SA	1	475.231
195	SA	1	475.233
196	SA	0.86	475.38
	NA_SA	0.12	477.351
197	SeAS_OC	0.87	475.371
	SeAS	0.12	477.349
198	OC	1	475.234
199	SeAS_OC	0.56	475.806
	OC	0.41	476.111
200	SA_SeAS_OC	0.43	476.078
	SA_OC	0.38	476.197
201	SA_MAD_SeAS_OC	0.23	476.701
	SA_SeAS_OC	0.21	476.815
	SA_MAD_OC	0.1	477.503
	MAD_SeAS_OC	0.09	477.683
	OC	0.07	477.923
	SA_OC	0.05	478.134
	MAD_OC	0.04	478.408
	SeAS_OC	0.04	478.563
	NA_SA_MAD_OC	0.03	478.616
	SA_MAD_SeAS	0.03	478.626
202	SSA	1	475.231
203	SSA	1	475.231
204	SSA	1	475.234
205	SA_SSA_MAD_SeAS_OC	0.22	476.768
	SA_SSA_SeAS_OC	0.19	476.871
	SA_SSA_MAD_OC	0.1	477.537
	SSA_MAD_SeAS_OC	0.08	477.709
	SSA_OC	0.08	477.73
	SA_SSA_OC	0.06	478.074
	SSA_MAD_OC	0.04	478.377
	SSA_SeAS_OC	0.04	478.388
	SA_SSA_MAD_SeAS	0.04	478.561
	NA_SA_SSA_MAD_OC	0.03	478.679
206	SA_SSA_MAD_SeAS_OC	0.22	476.764
	SA_SSA_SeAS_OC	0.19	476.918
	SSA_OC	0.11	477.457
	SA_SSA_MAD_OC	0.09	477.593
	SSA_MAD_SeAS_OC	0.09	477.668
	SA_SSA_OC	0.06	478.041
	SSA_SeAS_OC	0.06	478.13
	SSA_MAD_OC	0.04	478.382
207	EU+NA	1	475.233
208	EU+NA_SSA	0.98	475.25
209	EU+NA_SeAS_OC	0.73	475.541
	EU+NA_SeAS	0.23	476.717
210	EU+NA	0.63	475.69
	EU+NA_SSA	0.15	477.127

Node	Ancestral State	P	-LOG(L)
211	SA_EU+NA_SSA_MAD_SeAS_OC	0.12	477.337
	SA_EU+NA_SSA_SeAS_OC	0.11	477.424
	SSA	0.1	477.561
	SA_EU+NA_SSA_MAD_OC	0.07	477.959
	EU+NA_SSA_MAD_SeAS_OC	0.06	478.041
	EU+NA_SSA_SeAS_OC	0.04	478.441
	SA_EU+NA_SSA_OC	0.04	478.517
	SSA_OC	0.04	478.565
	EU+NA_SSA	0.03	478.729
	EU+NA_SSA_OC	0.03	478.789
	EU+NA_SSA_MAD_OC	0.03	478.869
	SA_SSA_SeAS_OC	0.02	479.247
	SA_EU+NA_MAD_SeAS_OC	0.02	479.293
	NA_SA_EU+NA_SSA_MAD_OC	0.02	479.297
212	EU+NA_SSA	0.96	475.273
213	SSA	0.79	475.463
	EU+NA_SSA	0.21	476.809
214	SSA	0.93	475.308
215	SSA_SeAS	0.87	475.368
216	SA_SSA	0.93	475.298
217	SA_EU+NA_SSA	0.93	475.307
218	SSA	0.52	475.885
	SSA_SeAS	0.13	477.273
	EU+NA_SSA	0.13	477.28
	SA_SSA	0.11	477.454
	SA_EU+NA_SSA	0.09	477.652
219	MAD	1	475.233
220	SSA_MAD	1	475.233
221	SSA_MAD	0.96	475.269
222	MAD	1	475.233
223	SSA_MAD	0.98	475.249
224	SSA	0.63	475.695
	SSA_MAD	0.37	476.223
225	SSA	0.8	475.46
226	SSA	0.87	475.37
227	NA	1	475.231
228	NA	1	475.231
229	NA	1	475.231
230	NA	1	475.231
231	NA	1	475.231
232	NA	1	475.231
233	NA	1	475.231
234	NA	1	475.231
235	NA	1	475.235
236	NA_SSA	0.94	475.289
237	NA	1	475.231
238	NA	1	475.231
239	NA	1	475.231
240	NA	0.83	475.422
	NA_SA	0.17	476.982
241	NA	0.98	475.25
242	NA	0.99	475.242
243	NA_SSA	0.95	475.286
244	SSA	0.73	475.55
	NA_SSA	0.25	476.612
245	SSA	0.17	477.03
	SA_EU+NA_SSA_SeAS_OC	0.08	477.724
	SA_EU+NA_SSA_MAD_SeAS_OC	0.08	477.8
	SA_EU+NA_SSA_MAD_OC	0.05	478.178
	EU+NA_SSA_MAD_SeAS_OC	0.05	478.253
	EU+NA_SSA_SeAS_OC	0.04	478.379
	SA_EU+NA_SSA_OC	0.04	478.508
	EU+NA_SSA_MAD_OC	0.03	478.843
	EU+NA_SSA_OC	0.02	478.934
246	SA	1	475.231
247	SA	1	475.231
248	SA	1	475.231
249	SA	1	475.231
250	SA	1	475.231
251	SA	1	475.231
252	NA_SA	1	475.236
253	SA	0.97	475.261
254	SA	0.99	475.24

Node	Ancestral State	P	-LOG(L)
255	SA	1	475.232
256	SA	1	475.231
257	SA	1	475.231
258	SA	1	475.231
259	SA	1	475.231
260	SA	1	475.231
261	SA	1	475.231
262	SA	1	475.231
263	NA	1	475.231
264	NA	1	475.231
265	NA_SA	1	475.232
266	NA_SA	0.9	475.336
267	NA_SA	1	475.236
268	NA_SA	1	475.236
269	SA	0.79	475.471
	NA_SA	0.21	476.784
270	SA	0.82	475.425
	NA_SA	0.18	476.964
271	SA	0.71	475.572
	NA_SA	0.26	476.576
272	NA_SA	0.99	475.237
273	SA	0.8	475.456
	NA_SA	0.2	476.832
274	SA	0.99	475.241
275	SA	1	475.231
276	SA	1	475.234
277	SA	1	475.231
278	SA	1	475.232
279	NA_SA	0.99	475.243
280	SA	0.88	475.358
281	SA	0.98	475.251
282	SA	0.99	475.236
283	SA	1	475.231
284	SA	1	475.231
285	SA	1	475.231
286	SA	1	475.231
287	SA	1	475.231
288	SA	1	475.234
289	SA	1	475.231
290	SA	1	475.231
291	NA_SA	1	475.232
292	NA_SA	0.87	475.374
	NA	0.13	477.249
293	SA	1	475.231
294	NA_SA	0.99	475.239
295	NA_SA	0.69	475.608
	SA	0.31	476.39
296	SA	0.6	475.74
	NA_SA	0.39	476.18
297	SA	0.84	475.405
	NA_SA	0.16	477.089
298	MAD_SeAS	0.96	475.274
299	SA_MAD_SeAS	0.6	475.748
	SA_SeAS	0.21	476.795
300	SeAS_OC	0.99	475.237
301	SA_MAD_SeAS	0.37	476.224
	SA_SeAS	0.17	476.976
	SA_MAD_SeAS_OC	0.13	477.284
	SeAS	0.06	478.121
302	SA_EU+NA_SSA_MAD_SeAS_OC	0.13	477.291
	SA_EU+NA_SSA_SeAS_OC	0.09	477.686
	EU+NA_SSA_MAD_SeAS_OC	0.05	478.232
	SA_SSA_MAD_SeAS_OC	0.05	478.257
	SA_EU+NA_SSA_MAD_OC	0.04	478.468
	SA_SSA_MAD_SeAS	0.04	478.492
	NA_SA_EU+NA_SSA_MAD_SeAS_OC	0.04	478.531
	SA_SSA_SeAS_OC	0.03	478.665
	EU+NA_SSA_SeAS_OC	0.03	478.687
	SA_EU+NA_SSA_MAD_SeAS	0.03	478.87
	NA_SA_EU+NA_SSA_SeAS_OC	0.02	478.93
	SA_SSA_SeAS	0.02	479.003
	SSA_MAD_SeAS_OC	0.02	479.139
	SA_EU+NA_SSA_OC	0.02	479.142

Node	Ancestral State	P	-LOG(L)
	SA_EU+NA_SSA_SeAS	0.02	479.171
303	NA_SA_EU+NA_SSA_MAD_SeAS_OC	0.08	477.812
	NA_SA_EU+NA_SSA_SeAS_OC	0.07	477.913
	NA_EU+NA_SSA_MAD_SeAS_OC	0.04	478.336
	NA_SA_SSA_MAD_SeAS_OC	0.04	478.408
	NA_EU+NA_SSA_SeAS_OC	0.04	478.479
	NA_SA_SSA_SeAS_OC	0.04	478.485
	NA_SA_EU+NA_SSA_MAD_OC	0.04	478.504
	NA_SA_SSA_MAD_SeAS	0.03	478.596
	NA_SA_SSA_SeAS	0.03	478.694
	NA_SA_EU+NA_SSA_MAD_SeAS	0.03	478.776
	NA_SA_EU+NA_SSA_OC	0.03	478.805
	NA_SSA_SeAS	0.03	478.819
	NA_SA_EU+NA_SSA_SeAS	0.03	478.832
	NA_SSA_SeAS_OC	0.03	478.844
	NA_SSA_MAD_SeAS_OC	0.03	478.885
	NA_SSA_MAD_SeAS	0.02	479.074
	NA_SA_SSA_MAD_OC	0.02	479.096
	NA_EU+NA_SSA_MAD_OC	0.02	479.195
	NA_EU+NA_SSA_SeAS	0.02	479.257
	NA_EU+NA_SSA_MAD_SeAS	0.02	479.265
	NA_SA_SSA_OC	0.02	479.281
	NA_SA_SSA_MAD	0.02	479.429
	NA_SA_EU+NA_MAD_SeAS_OC	0.01	479.433
	NA_SA_SSA	0.01	479.479
	NA_SA_EU+NA_SSA_MAD	0.01	479.481
	NA_SA_EU+NA_SeAS_OC	0.01	479.543
	NA_SSA_MAD_OC	0.01	479.637
	NA_SA_EU+NA_SSA	0.01	479.688
	NA_EU+NA_SSA_OC	0.01	479.714
304	NA_SA_EU+NA_SSA_SeAS_OC	0.05	478.184
	NA_SA_SSA_SeAS	0.05	478.294
	NA_SA_SSA_SeAS_OC	0.05	478.297
	NA_EU+NA_SSA_SeAS_OC	0.04	478.396
	NA_SA_EU+NA_SSA_MAD_SeAS_OC	0.04	478.42
	NA_SSA_SeAS	0.04	478.426
	NA_SSA_SeAS_OC	0.04	478.456
	NA_SA_SSA_MAD_SeAS	0.04	478.47
	NA_SA_SSA_MAD_SeAS_OC	0.04	478.509
	NA_EU+NA_SSA_MAD_SeAS_OC	0.04	478.518
	NA_SA_EU+NA_SSA_SeAS	0.04	478.581
	NA_SSA_MAD_SeAS_OC	0.03	478.622
	NA_SSA_MAD_SeAS	0.03	478.623
	NA_SA_EU+NA_SSA_MAD_SeAS	0.03	478.787
	NA_EU+NA_SSA_SeAS	0.03	478.837
	NA_EU+NA_SSA_MAD_SeAS	0.02	478.931
	NA_SA_EU+NA_SeAS_OC	0.02	479.066
	NA_SA_EU+NA_MAD_SeAS_OC	0.02	479.215
	NA_SeAS	0.02	479.274
	NA_SA_SeAS_OC	0.02	479.301
	NA_SA_SeAS	0.02	479.359
	NA_EU+NA_SeAS_OC	0.02	479.384
	NA_SA_MAD_SeAS_OC	0.02	479.405
	NA_EU+NA_MAD_SeAS_OC	0.02	479.406
	NA_SA_MAD_SeAS	0.01	479.487
	NA_SeAS_OC	0.01	479.563
	NA_SA_EU+NA_SeAS	0.01	479.584
	NA_MAD_SeAS_OC	0.01	479.633
	NA_SA_EU+NA_MAD_SeAS	0.01	479.676
	NA_MAD_SeAS	0.01	479.694
	SA_EU+NA_SSA_SeAS_OC	0.01	479.757
	SA_EU+NA_SSA_MAD_SeAS_OC	0.01	479.858
	NA_EU+NA_MAD_SeAS	0.01	479.939
	NA_EU+NA_SeAS	0.01	479.952
	SA_SSA_SeAS_OC	0.01	480.021
	SA_SSA_MAD_SeAS_OC	0.01	480.065
	EU+NA_SSA_MAD_SeAS_OC	0.01	480.09
	EU+NA_SSA_SeAS_OC	0.01	480.129
	SA_SSA_MAD_SeAS	0.01	480.159
	SA_SSA_SeAS	0.01	480.171

Table S2. Alternative ancestral climatic niche reconstructions. P: probability, L: likelihood. States: TE (Temperate), TD (Tropical season dry forest, savannah, scrubland), TW (Tropical moist/wet forest)

Node	Ancestral State	P	-LOG(L)
1	TE_TW	1	-268.787
2	TE_TW	0.84	-268.961
	TE	0.16	-270.621
3	TE_TW	0.85	-268.952
4	TE	1	-268.787
5	TE	1	-268.787
6	TE	0.61	-269.277
	TE_TW	0.33	-269.896
7	TE	1	-268.787
8	TE	1	-268.787
9	TE	0.78	-269.03
	TE_TW	0.18	-270.515
10	TE_TD	1	-268.789
11	TE_TD	0.5	-269.477
	TE	0.5	-269.483
12	TE	0.72	-269.122
	TE_TD	0.18	-270.502
13	TE_TD	1	-268.787
14	TE_TD	0.81	-268.998
	TD	0.19	-270.45
15	TE_TD	0.74	-269.083
	TD	0.26	-270.15
16	TE_TD_TW	0.73	-269.103
	TD_TW	0.26	-270.127
17	TE_TD	0.38	-269.744
	TE_TD_TW	0.32	-269.934
	TD	0.17	-270.579
	TD_TW	0.13	-270.818
18	TE	1	-268.787
19	TE	0.49	-269.505
	TE_TD	0.29	-270.012
	TE_TW	0.14	-270.76
	TE_TD_TW	0.08	-271.353
20	TE	0.5	-269.481
	TE_TD	0.21	-270.343
	TE_TW	0.17	-270.583
21	TD	1	-268.787
22	TD_TW	1	-268.787
23	TD_TW	0.65	-269.218
	TD	0.35	-269.837
24	TD_TW	0.81	-268.999
	TW	0.19	-270.442
25	TD_TW	0.6	-269.3
	TD	0.36	-269.815
26	TD	1	-268.787
27	TD	1	-268.787
28	TD	0.54	-269.397
	TD_TW	0.46	-269.573
29	TE_TW	1	-268.787
30	TE_TW	0.79	-269.02
	TE	0.21	-270.359
31	TE	1	-268.787
32	TE_TW	0.72	-269.116
	TE	0.28	-270.059
33	TW	1	-268.787

Node	Ancestral State	P	-LOG(L)
34	TW	1	-268.787
35	TE_TW	0.7	-269.139
	TW	0.3	-270.005
36	TD_TW	0.36	-269.807
	TE_TD_TW	0.29	-270.029
	TW	0.16	-270.628
	TE_TD	0.12	-270.88
	TE_TW	0.07	-271.476
37	TE_TW	0.24	-270.229
	TE_TD_TW	0.18	-270.48
	TE_TD	0.14	-270.726
	TW	0.14	-270.748
	TD	0.14	-270.765
	TD_TW	0.09	-271.142
	TE	0.06	-271.576
38	TW	1	-268.787
39	TW	1	-268.787
40	TD_TW	1	-268.787
41	TW	0.65	-269.211
	TD_TW	0.35	-269.85
42	TW	0.94	-268.846
43	TW	0.98	-268.809
44	TW	1	-268.787
45	TW	1	-268.787
46	TD_TW	1	-268.79
47	TW	0.5	-269.478
	TD_TW	0.5	-269.49
48	TD_TW	1	-268.788
49	TD_TW	0.42	-269.646
	TE_TW	0.25	-270.172
	TW	0.18	-270.502
	TE_TD_TW	0.14	-270.718
50	TD_TW	0.33	-269.904
	TW	0.31	-269.963
	TE_TW	0.25	-270.174
	TE_TD_TW	0.11	-270.955
51	TE_TW	0.57	-269.347
	TE_TD_TW	0.41	-269.686
52	TD_TW	0.52	-269.438
	TW	0.32	-269.919
	TE_TD_TW	0.07	-271.388
53	TD	1	-268.789
54	TD_TW	0.84	-268.962
55	TW	1	-268.788
56	TD_TW	0.82	-268.989
	TW	0.15	-270.704
57	TD	1	-268.787
58	TD_TW	1	-268.787
59	TD	0.88	-268.92
	TD_TW	0.12	-270.873
60	TD_TW	1	-268.787
61	TD	0.7	-269.147
	TD_TW	0.29	-270.033
62	TD	1	-268.787
63	TD_TW	1	-268.789
64	TD	0.69	-269.152
	TD_TW	0.31	-269.973
65	TD	0.72	-269.114
	TD_TW	0.21	-270.342
66	TD_TW	0.58	-269.329

Node	Ancestral State	P	-LOG(L)
	TD	0.22	-270.287
	TW	0.17	-270.563
67	TD_TW	0.34	-269.854
	TW	0.27	-270.114
	TE_TD_TW	0.15	-270.687
	TD	0.12	-270.887
	TE_TW	0.09	-271.251
68	TD_TW	0.52	-269.436
	TW	0.48	-269.527
69	TW	1	-268.787
71	TW	0.53	-269.426
	TD_TW	0.47	-269.538
71	TD_TW	0.99	-268.802
72	TW	1	-268.787
73	TW	1	-268.787
74	TW	1	-268.787
75	TW	0.62	-269.258
	TD_TW	0.38	-269.766
76	TD_TW	1	-268.789
77	TW	0.55	-269.379
	TD_TW	0.42	-269.664
78	TD	1	-268.787
79	TD	1	-268.787
80	TD	1	-268.787
81	TD	1	-268.788
82	TD_TW	1	-268.788
83	TW	0.53	-269.416
	TD_TW	0.46	-269.569
84	TW	0.76	-269.059
	TD_TW	0.24	-270.227
85	TD_TW	1	-268.789
86	TD_TW	0.82	-268.984
	TD	0.18	-270.512
87	TD_TW	0.78	-269.041
	TW	0.15	-270.656
88	TD	1	-268.787
89	TD	1	-268.787
90	TD	1	-268.787
91	TD	1	-268.787
92	TD	1	-268.787
93	TD_TW	0.74	-269.091
	TD	0.26	-270.132
94	TD_TW	1	-268.788
95	TD_TW	0.65	-269.215
	TW	0.19	-270.425
	TD	0.15	-270.661
96	TW	0.52	-269.443
	TD_TW	0.37	-269.787
97	TW	1	-268.787
98	TW	1	-268.787
99	TW	1	-268.787
100	TW	1	-268.787
101	TW	1	-268.787
102	TD_TW	1	-268.787
103	TW	0.86	-268.942
	TD_TW	0.14	-270.729
104	TW	1	-268.787
105	TW	1	-268.787
106	TW	1	-268.787
107	TW	1	-268.787

Node	Ancestral State	P	-LOG(L)
108	TW	0.96	-268.828
109	TW	1	-268.787
110	TW	1	-268.787
111	TW	1	-268.791
112	TW	1	-268.787
113	TW	1	-268.787
114	TW	1	-268.787
115	TW	1	-268.787
116	TW	1	-268.787
117	TW	1	-268.787
118	TW	1	-268.787
119	TW	1	-268.787
120	TD_TW	1	-268.787
121	TD_TW	0.7	-269.14
	TW	0.3	-270.001
122	TW	0.8	-269.012
	TD_TW	0.2	-270.39
123	TW	1	-268.787
124	TW	0.99	-268.799
125	TW	1	-268.789
126	TW	1	-268.789
127	TW	0.84	-268.966
	TD_TW	0.14	-270.724
128	TD_TW	0.99	-268.794
129	TW	0.85	-268.949
	TD_TW	0.14	-270.753
130	TD	1	-268.787
131	TD	1	-268.788
132	TD_TW	1	-268.788
133	TW	1	-268.787
134	TD_TW	0.85	-268.948
	TW	0.15	-270.693
135	TD_TW	1	-268.789
136	TD	0.57	-269.35
	TD_TW	0.43	-269.63
137	TD	0.7	-269.146
	TD_TW	0.3	-269.986
138	TD_TW	0.57	-269.35
	TD	0.4	-269.693
139	TD	0.51	-269.46
	TD_TW	0.49	-269.501
140	TD	1	-268.787
141	TD	0.54	-269.409
	TD_TW	0.46	-269.557
142	TW	1	-268.787
143	TW	1	-268.787
144	TW	1	-268.787
145	TW	1	-268.787
146	TW	1	-268.787
147	TW	1	-268.787
148	TD_TW	0.97	-268.813
149	TD	1	-268.787
150	TD	1	-268.787
151	TD_TW	0.95	-268.84
152	TW	0.91	-268.881
153	TW	1	-268.787
154	TW	1	-268.787
155	TW	0.97	-268.82
156	TD_TW	1	-268.788
157	TD_TW	1	-268.787

Node	Ancestral State	P	-LOG(L)
158	TW	0.59	-269.312
	TD_TW	0.4	-269.692
159	TW	0.93	-268.857
160	TW	1	-268.787
161	TW	0.98	-268.812
162	TW	1	-268.787
163	TW	1	-268.787
164	TW	1	-268.787
165	TW	0.98	-268.803
166	TW	0.98	-268.81
167	TD_TW	0.99	-268.796
168	TW	0.82	-268.983
	TD_TW	0.17	-270.533
169	TW	1	-268.787
170	TW	1	-268.787
171	TW	1	-268.787
172	TW	1	-268.787
173	TW	1	-268.787
174	TW	1	-268.787
175	TW	1	-268.787
176	TW	1	-268.787
177	TW	1	-268.787
178	TW	1	-268.787
179	TW	1	-268.787
180	TW	1	-268.787
181	TW	1	-268.787
182	TW	1	-268.787
183	TW	1	-268.787
184	TW	1	-268.787
185	TW	1	-268.787
186	TW	1	-268.787
187	TD_TW	0.98	-268.81
188	TW	1	-268.787
189	TW	1	-268.787
190	TW	1	-268.787
191	TW	1	-268.787
192	TW	1	-268.787
193	TW	1	-268.787
194	TW	1	-268.787
195	TW	1	-268.787
196	TW	1	-268.787
197	TW	1	-268.787
198	TW	1	-268.787
199	TW	1	-268.787
200	TW	1	-268.787
201	TW	0.89	-268.901
202	TW	1	-268.787
203	TW	1	-268.787
204	TW	1	-268.787
205	TW	0.95	-268.834
206	TW	0.98	-268.812
207	TD	1	-268.787
208	TD	1	-268.79
209	TW	1	-268.787
210	TD_TW	0.98	-268.804
211	TW	0.63	-269.256
	TD_TW	0.37	-269.783
212	TD	1	-268.787
213	TD	1	-268.787
214	TD	1	-268.787

Node	Ancestral State	P	-LOG(L)
215	TD	1	-268.787
216	TD	1	-268.787
217	TD	1	-268.787
218	TD	1	-268.787
219	TD	1	-268.787
220	TD	1	-268.787
221	TD	1	-268.787
222	TD	1	-268.787
223	TD	1	-268.787
224	TD	1	-268.787
225	TD	1	-268.787
226	TD	1	-268.787
227	TD	1	-268.787
228	TD	1	-268.787
229	TD	1	-268.787
230	TD	1	-268.787
231	TD	1	-268.787
232	TD	1	-268.787
233	TD	1	-268.787
234	TD	1	-268.787
235	TD	1	-268.787
236	TD	1	-268.788
237	TD	1	-268.787
238	TD	1	-268.787
239	TD	1	-268.787
240	TD	0.76	-269.065
	TD_TW	0.24	-270.204
241	TD	0.92	-268.875
242	TD	0.93	-268.865
243	TD	0.94	-268.852
244	TD_TW	0.95	-268.838
245	TD_TW	0.62	-269.258
	TW	0.36	-269.815
246	TW	1	-268.787
247	TW	1	-268.787
248	TW	0.81	-269.003
	TD_TW	0.19	-270.426
249	TW	0.96	-268.824
250	TW	1	-268.787
251	TW	0.99	-268.796
252	TW	0.83	-268.969
	TD_TW	0.17	-270.581
253	TW	0.99	-268.792
254	TW	1	-268.789
255	TW	1	-268.788
256	TW	1	-268.787
257	TW	1	-268.787
258	TW	1	-268.787
259	TD_TW	1	-268.787
260	TW	0.83	-268.98
	TD_TW	0.17	-270.53
261	TW	0.95	-268.84
262	TW	1	-268.791
263	TW	1	-268.787
264	TW	1	-268.787
265	TW	1	-268.787
266	TW	1	-268.787
267	TW	1	-268.787
268	TW	0.85	-268.951
	TD_TW	0.15	-270.675

Node	Ancestral State	P	-LOG(L)
269	TW	1	-268.792
270	TW	1	-268.79
271	TW	1	-268.788
272	TW	1	-268.787
273	TW	1	-268.787
274	TW	1	-268.787
275	TW	1	-268.787
276	TW	1	-268.787
277	TW	1	-268.787
278	TW	1	-268.787
279	TW	1	-268.787
280	TW	1	-268.787
281	TW	1	-268.787
282	TW	1	-268.787
283	TW	1	-268.787
284	TW	1	-268.787
285	TW	1	-268.787
286	TW	1	-268.787
287	TW	1	-268.787
288	TW	1	-268.787
289	TW	1	-268.787
290	TW	1	-268.787
291	TW	1	-268.787
292	TW	1	-268.787
293	TW	1	-268.787
294	TW	1	-268.787
295	TW	1	-268.787
296	TW	1	-268.787
297	TW	1	-268.787
298	TW	1	-268.787
299	TW	1	-268.787
300	TW	1	-268.787
301	TW	1	-268.787
302	TD_TW	0.62	-269.27
	TW	0.38	-269.748
303	TD_TW	0.8	-269.016
	TD	0.2	-270.412
304	TD_TW	0.76	-269.067
	TW	0.22	-270.324
